# Constitutive expression of an A-5 subgroup member in the DREB transcription factor subfamily from *Ammopiptanthus mongolicus* enhanced abiotic stress tolerance and anthocyanin accumulation in transgenic Arabidopsis

**DOI:** 10.1371/journal.pone.0224296

**Published:** 2019-10-23

**Authors:** Meiyan Ren, Zhilin Wang, Min Xue, Xuefeng Wang, Feng Zhang, Yu Zhang, Wenjun Zhang, Maoyan Wang

**Affiliations:** College of Life Sciences, Inner Mongolia Agricultural University, Hohhot, China; National Taiwan University, TAIWAN

## Abstract

Dehydration-responsive element-binding (DREB) transcription factors (TFs) are key regulators of stress-inducible gene expression in plants. Anthocyanins, an important class of flavonoids, protect plants from reactive oxygen species produced under abiotic stresses. However, regulation of DREBs on anthocyanin accumulation is largely unknown. Here, an A-5 subgroup DREB gene (*AmDREB3*) isolated from *Ammopiptanthus mongolicus*, a desert broadleaf shrub with very high tolerance to harsh environments, was characterized in terms of both abiotic stress tolerance and anthocyanin accumulation. AmDREB3 does not contain the transcriptional repression motif EAR, and the protein was located in the nucleus and has transcriptional activation capacity. The transcription of *AmDREB3* was differentially induced in the shoots and roots of *A*. *mongolicus* seedlings under drought, salt, heat, cold, ultraviolet B, and abscisic acid treatments. Moreover, the transcript levels in twigs, young leaves, and roots were higher than in other organs of *A*. *mongolicus* shrubs. Constitutively expressing *AmDREB3* improved the tolerance of transgenic Arabidopsis to drought, high salinity and heat, likely by inducing the expression of certain stress-inducible genes. The transgenic Arabidopsis seedlings also exhibited an obvious purple coloration and significant increases in anthocyanin accumulation and/or oxidative stress tolerance under drought, salt, and heat stresses. These results suggest that the AmDREB3 TF may be an important positive regulator of both stress tolerance and anthocyanin accumulation.

## Introduction

Drought, high salinity, and extreme temperatures are major abiotic stressors that significantly affect the geographical distribution of plants in nature and limit crop productivity in agriculture [1]. Plants have developed the capacity to respond and tolerate to these stressors by altering processes at physiological and molecular levels. Molecular alterations usually include the perception and transduction of stress signals by plant cells and prompt the activation of downstream transcription factors (TFs) on stress signaling pathways. The TFs directly trigger the expression of an array of functional genes, which in turn produces protective mechanisms in plants [1–2]. Among various stress-related TFs, the dehydration responsive element binding (DREB) TFs have a major role in plant response to abiotic stressors through regulatory networks, and some DREB genes have been shown to be good candidates for genetic engineering to breed stress-tolerant crops [1,3].

The DREB TFs constitute one of four major subfamilies in the plant-specific APETALA2/ethylene responsive factor (AP2/ERF) family in Arabidopsis (*Arabidopsis thaliana*) and the DREB subfamily can be further divided into six subgroups, A-1 to A-6 [4]. The genes for A-1 and A-2 subgroups in Arabidopsis, generally known as *DREB1s*/*CBFs* and *DREB2s*, respectively, and their orthologous genes in other plants have been extensively studied and found to be closely involved in the response of plants to abiotic stressors compared to genes in the other four subgroups [1,3,5]. Information about members in the other subgroups remains limited.

The A-5 subgroup in Arabidopsis consists of 16 members and only the regulatory function of *AtRAP2*.*1* has been investigated so far [4]. *AtRAP2*.*1* was strongly induced by drought and low temperature through an ABA-independent pathway. However, the AtRAP2.1 protein repressed the transcription of several dehydration-responsive element (DRE)-mediated genes and negatively regulated plant tolerance to cold and drought stressors [6]. *GhDBP1* and *MsDREBA5* are orthologous genes of *AtRAP2*.*1* in cotton (*Gossypium hirsutum*) and *Malus sieversii* Roem., respectively, and were induced by high salinity and drought and acted as repressors of some DRE-mediated gene transcription and/or salt tolerance [7–9]. In contrast, other A-5 subgroup genes from different plant species, including *GmDREB1*, *GmDREB*2, and *GmDREB*3 from soybean (*Glycine max*), *StDREB2* from potato (*Solanum tuberosum*), *PeDREB2a* from *Populus euphratic*, *HhDREB2* from *Halimodendron halodendron*, as well as those from mosses, including *Physcomitrella patens* and *Syntrichia caninervis*, played important positive regulatory roles in both stress-inducible gene expression and/or stress tolerance improvement of their transgenic plants [10–17]. There is thus evident functional divergence of the A-5 subgroup genes in different species. Therefore, it is necessary to investigate more members of this subgroup in order to elucidate their function and regulation mechanisms in various species, especially those with a high tolerance to harsh environments.

Anthocyanins, a main class of flavonoids, serve as important protectants against excessive reactive oxygen species (ROS) produced under abiotic stresses, in addition to their roles in plant development [18]. The anthocyanin biosynthetic pathway is a major branch of the general phenylpropanoid pathway and is controlled by a series of enzymes [19]. Numerous studies have indicated that the production of anthocyanins can be induced by various abiotic stressors such as ultraviolet-B (UV-B) radiation, drought, salinity, and extreme temperatures. A high level of anthocyanins has been recognized to protect plant cells from oxidative stress through scavenging excessive ROS produced under stress conditions [19–20]. The biosynthesis of anthocyanins is also regulated by many factors in plant cells, such as some members in the MYB, bHLH, WD40, and NAC TF families [19, 21–22]. In particular, recent studies revealed that a ternary WD40-bHLH-MYB (WBM) TF complex and its regulatory miRNAs are master positive regulators controlling the synthesis process. The WBM complexes were found to primarily regulate the late steps of the anthocyanin-specific biosynthetic pathway [23–25], while the miRNAs (mainly *miR828*, *miR156*, and *TAS4*) regulated expression of the members in the WBM complex [19, 26–28]. Several reports have also described positive or negative regulation of *DREB* heterologous expression on anthocyanin accumulation under cold and/or drought stresses. Such *DREBs* include *PpCBF1* from peach (*Prunus persica*), *EguCBF1s* from *Eucalyptus gunnii*, *RdreB1BI* (codon-optimized *OsDREB1B*) from rice, and *AtCBF1* and *AtCBF3* from *A*. *thaliana*, all which belong to the A-1 subgroup [29–34]. However, the effects and regulatory mechanisms of DREBs on anthocyanin accumulation under different abiotic stresses are largely unknown.

*Ammopiptanthus mongolicus* is the only evergreen broadleaf relic shrub growing in the central Asian desert where the climate is dry year-round, seasonally cold or hot, and intensely UV-radiated. The soils in the habitats are poor and highly saline. Thus, *A*. *mongolicus* has a very high tolerance to abiotic stressors and is increasingly used as a model plant for studying stress tolerance [35–37]. So far, only one *DREB*, *AmDREB2C*, has been functionally characterized in *A*. *mongolicus*, which enhanced freezing, drought, and heat tolerances and regulated fatty acid composition in transgenic Arabidopsis [37]. Here, we cloned and investigated the function of another *DREB*, *AmDREB3*, from *A*. *mongolicus*. The gene showed universally increased transcription in lab-grown seedlings under controlled abiotic stress and UV-B and ABA treatments. Constitutively expressing *AmDREB3* in Arabidopsis enhanced the tolerances to drought, salt, and heat stressors by increasing the expression of some stress-inducible genes. Constitutive expression of *AmDREB3* also enhanced anthocyanin accumulation and oxidative stress tolerance in transgenic seedlings under stress conditions. These findings provide new evidence of regulatory roles of DREB TFs in both stress tolerance and anthocyanin accumulation.

## Materials and methods

### Stress treatments and field sampling of *A*. *mongolicus*

Seeds of *A*. *mongolicus* were surface-sterilized with 1% (v/v) sodium hypochlorite and cultured in pots filled with sand under normal conditions as described previously [35]. After culturing for one month, seedlings were subjected to drought, salt, heat, cold, UV-B or ABA treatment. Briefly, the drought treatment was conducted by stopping watering of the seedlings for 14 d. The seedlings were collected separately as shoots and roots at 0 (control), 4, 6, 8, 10, 11, 12, 13, and 14 d after watering was stopped. For the UV-B treatment, seedlings were exposed to artificial UV-B radiation (40 w) for 48 h. The ABA treatment was performed as follows: seedlings were washed with tap water after they were carefully removed from the sand and then cultured in an aerated Hoagland nutrient solution with 100 μM (±)-*cis*, *trans*-ABA after a one-day adaptive culture in aerated Hoagland solution. The salt, cold and heat treatments were carried out as described by Yin et al. [37]. Except for the drought treatment, all treated seedlings were harvested separately as shoots and roots at 0 (control), 2, 6, 12, 24, or 48 h after the initiation of each treatment.

Various tissue samples were also collected for gene expression analysis from naturally-grown *A*. *mongolicus* shrubs in the southern suburb of Hohhot, Inner Mongolia, China (111.8 E and 40.4 N), which was permitted by the private company (Inner Mongolia M-Grass Ecology and Environment (Group) Co., Ltd.). Flower buds were sampled in late April 2017, and other tissues were sampled in late May 2017. This filed study did not involve endangered or protected species.

All samples were immediately frozen in liquid nitrogen and then stored at -76°C for RNA extraction.

### Real-time quantitative PCR

Real-time quantitative PCR (RT-qPCR) was used to examine the transcription of *AmDREB3* under different conditions and in different organs. Total RNAs were isolated from the stored (-76°C) *A*. *mongolicus* samples using a modified Trizol method [35] and genomic DNA contaminants were removed with RQⅠ DNase treatment. First-strand cDNAs were synthesized using the purified RNAs as templates with M-MLV reverse transcriptase (Takara Biotech, Dalian, China) and were diluted 10-fold with deionized water and then used as templates for PCR. The *A*. *mongolicus* a-tubulin gene (*AmTUB*) was used as an internal control. The primers for the PCR are listed in S1 Table. The SYBR^®^ Green PCR Master Mix (Takara Biotech, Dalian, China) was used for PCR. Real-time PCR reactions were performed on the LightCycler^®^ 480 System. The PCR cycling program was as follows: pre-incubation at 94°C for 1 min, followed by amplification of 40 cycles at 95°C for 15 sec, 62°C for 15 sec, and 72°C for 30 sec, with a final melting step at 95°C for 15 sec and 60°C for 1 min. All the PCR reactions were performed at least three times for each RNA sample. Each sample was run in triplicate. Relative transcript abundance was calculated using the relative 2^–ΔΔCt^ method [38].

### Gene cloning and protein prediction

The coding region cDNA and gDNA (extracted with the sodium dodecyl sulfate method) of *AmDREB3* were amplified by PCR using the specific primer pair *AmDREB3-2* (containing the *Bgl*Ⅱ and *Bst*EⅡ sites) listed in S1 Table and then sequenced. Peptide sequences and alignments were predicted using ClustalX 2.1 and the GeneDoc 2.6 program. The phylogenetic tree of protein sequences was constructed using the MEGA7 program [39]. The distances between branches were calculated using the Neighbor-joining method with 1,000 bootstrap samples. The nuclear location signal (NLS) sequence was predicted using the tools on http://nls-mapper.iab.keio.ac.jp/cgi-bin/NLS_Mapper_form.cgi.

### Subcellular localization analysis

The coding region cDNA of *AmDREB3* excluding the termination codon was amplified by PCR from this genes’ cloning vector DNA using the primer pair *AmDREB3-3* (S1 Table). The PCR product was inserted upstream of a green fluorescent protein (GFP) coding region in the pBI-GFP vector. Isolation and transfection of Arabidopsis leaf protoplasts were performed as previously described [40], and the transfection products were observed under fluorescence microscopy (Nikon NT88-V3, Japan).

### Transactivation analysis

The cDNA of coding region of *AmDREB3* was amplified using the specific primer pair *AmDREB3-4* (S1 Table). The PCR product was cloned into the DNA-binding domain vector pGBKT7, a yeast expression vector with the promoter and terminator of the *ADH1* gene, to construct the pGBKT7-*AmDREB3* fusion plasmid. The recombinant plasmid was then transferred into the yeast strain Y2HGold carrying the reporter genes *His3* and *MEL1*. This yeast strain cannot grow on the synthetic dropout (SD) media plates without histidine (His) or tryptophan (Trp), which cannot induce α-galactosidase (α-Gal) activity. The transformed yeast cells were plated onto SD plates without Trp or His. The plates were incubated at 30°C for 3 d and an X-α-Gal assay was used to examine the transactivation ability of AmDREB3.

### Construction and stress tolerance analyses of transgenic Arabidopsis

The cDNA of *AmDREB3* coding region was inserted downstream of the cauliflower mosaic virus 35S (CaMV35S) promoter in plant expression vector pCAMBIA3301 (p3301) with a selectable marker gene for phosphinothricin (PPT). The recombinant plasmid was verified by sequencing and introduced into *Agrobacterium tumefaciens* GV3101 cells using the freeze-thaw method and subsequently introduced into *A*. *thaliana* by the floral dip method [41]. The T_1_ seedlings were screened by PPT and then the transgene in the genomes of surviving seedlings was detected by PCR using the primer pair *AmDREB3-2* (S1 Table). The T_2_ lines with single copy integration (with a 3:1 separation ratio of green:white seedlings) and high transcript level of the transgene selected using semi-quantitative RT-PCR were used to produce T_3_ seeds. Then the homozygotes showing 100% PPT resistance were used for stress tolerance analyses. The long-day condition (16-h-light/8-h-dark cycle) at 22°C was considered normal conditions.

Drought tolerance at the seed germination stage was tested on 1/2 MS agar plates containing 300 or 350 mM mannitol [37]. The germination rates (emergence of radicles) and root lengths of the seedlings were measured at 3 d and 10 d of the incubation period, respectively. At the seedling stage, drought tolerance was evaluated by leaving ten-day-old seedlings to be unwatered for 17 d, and then the seedlings were returned to their regular watering schedule of once every 5 d with tap water (the same watering frequency was used for all regular watering schedule below). Survival rates were determined a week later. The salt treatment was carried out by watering ten-day-old seedlings with a 300 mM NaCl solution once and then returning the seedlings to their regular watering schedule. Fresh weights of the seedlings were weighed a week after the treatment. For the heat stress treatment, ten-day-old seedlings were exposed to 37°C for 1 h, then were returned to their previous normal conditions for recovery for 2 h, and lastly were treated at 53°C for 2 h. The treatments at 37°C and 53°C were conducted by placing the pots in a plastic tray containing water to avoid drought stress. Plant heights and weights were measured on the 14th day of the recovery period under normal conditions. For cold tolerance analysis, two-week-old seedlings removed from 1/2 MS agar plates were put on filter paper saturated with water and exposed to -6°C for 1 h, and then allowed to recover at 22°C for 24 h [37]. At the end of the recovery, survival rates were measured. ABA sensitivity at the seed germination stage was tested on 1/2 MS agar plates containing 0.75, 1.00 or 1.5 μM ABA.

All these assays were repeated at least three times, with each line containing approximately 60 seeds or 25 seedlings per replicate. Wild-type Arabidopsis was used as a control in all of the assays.

### Measurement of physiological indices

Three-week-old seedlings grown in pots were stopped watering or were watered with a 300 mM NaCl solution. At 10 d or 5 d after the drought or salt treatment, anthocyanin contents were measured according to the method described by Zhang et al. [42]. The contents of hydrogen peroxide (H_2_O_2_) and malondialdehyde (MDA) as well as the activities of catalases (CATs), peroxidases (PODs), and superoxide dismutases (SODs) were measured using spectrophotometry with detection kits (Keming, Suzhou, China) in accordance with the manufacturer’s instructions.

Water loss was measured by placing detached shoots from three-week-old seedlings on petri dishes for dehydration at 25°C and weighing them every 0.5 h till to 7 h. Water loss was calculated as: 1- (FW_0_–FW_t_)/FW_0_×100%, where FW_0_ is the fresh weight at 0 h and FW_t_ is the fresh weight at the indicated time point.

Each index was measured based on three independent biological replicates.

### Expression analyses of stress-responsive genes in transgenic Arabidopsis

The genes used for expression analyses in transgenic Arabidopsis are listed in S1 Table. Two-week-old seedlings grown on 1/2 MS agar plates under normal conditions were used as control samples. The remaining same-aged seedlings were submerged in a solution containing 350 mM mannitol (to induce osmotic stress) or 175 mM NaCl (considered as salt stress), or were exposed to heat stress (37°C) for 5 h. Then, the seedlings were collected and stored at -76°C after being immediately frozen in liquid nitrogen.

Total RNAs extracted from the stored (-76°C) samples were purified and used to examine the gene expression levels by semi-quantitative RT-PCR. Arabidopsis *ACTIN2* was used as an internal reference gene. All the PCR reactions were performed three to four times for each RNA sample.

### Promoter analysis

The promoter region of *AmDREB3* was cloned using the Genome Walking Kit (TaKaRa, Dalian, China). The primers used are listed in S1 Table. Promoter sequence was analyzed using the PLACE Signal Scan Search Program (http://www.dna.affrc.go.jp/PLACE/signalscan.html).

## Results

### Sequence features and phylogenetic analysis of AmDREB3

**Fig 1:**
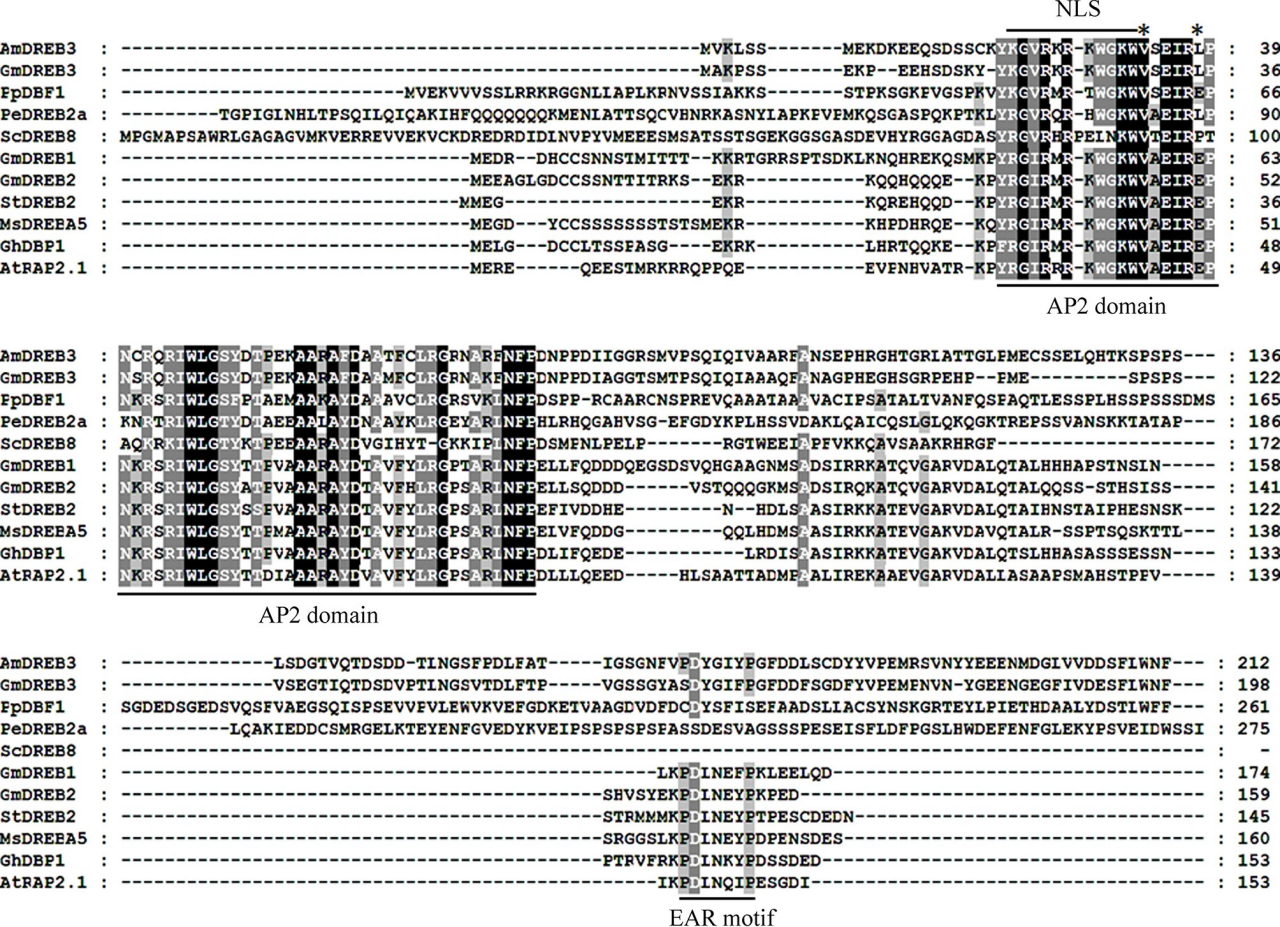
Multiple sequence alignments of 10 DREBs belonging to the A-5 subgroup from different plant species. The amino acid residues with 100% and more than 80% or 60% conservation among all sequences are indicated in white on a black or grey background or in black on a grey background, respectively. The AP2/ERF DNA-binding domain, NLS, and EAR motif are indicated. The asterisks indicate the 14th V and the 19th L residues inside the AP2/ERF domain. The accession numbers of all protein sequences are listed in S2 Table.

The full-length cDNA sequence of *AmDREB3* was identified in *A*. *mongolicus* in our previous study [34]. The sequence analysis of full-length cDNA and gDNA indicated that *AmDREB3* is intronless. The protein encoded by *AmDREB3* has the highest sequence similarity to GmDREB3 (identity = 69.01%) among all DREB sequences in GenBank. AmDREB3 consists of 212 amino acids and was predicted to contain a conserved AP2/ERF DNA-binding domain of 58 amino acids (between 20 to 77 residues) at its N-terminal region. Multiple sequence alignments revealed that the AP2/ERF domain contains the conserved 14th Val (V) as do all other A-5 subgroup members, but the conserved 19th Glu (E) was replaced by a Leu (L) residue as in the GmDREB3 and PeDREB2a proteins (Fig 1). Moreover, a putative NLS (KGVRKRKWGKW, 22–33) exists in the N-terminal region of the AP2/ERF domain. However, the ERF-associated amphiphilic repression (EAR)-motif (DLNxxP) that appears in the C-terminal regions of most A-5 subgroup DREB proteins changes to DYGIYP in the AmDREB3 protein (Fig 1).

Phylogenetic analysis using 39 DREB proteins from 11 species showed that AmDREB3 was clustered in the same clade with all other A-5 subgroup members, such as soybean GmDREB1 and GmDREB2, cotton GhDBP1, and potato StDREB2, having the closest relationship with GmDREB3 (S1 Fig).

### Subcellular localization and transactivation ability of the AmDREB3 protein

**Fig 2:**
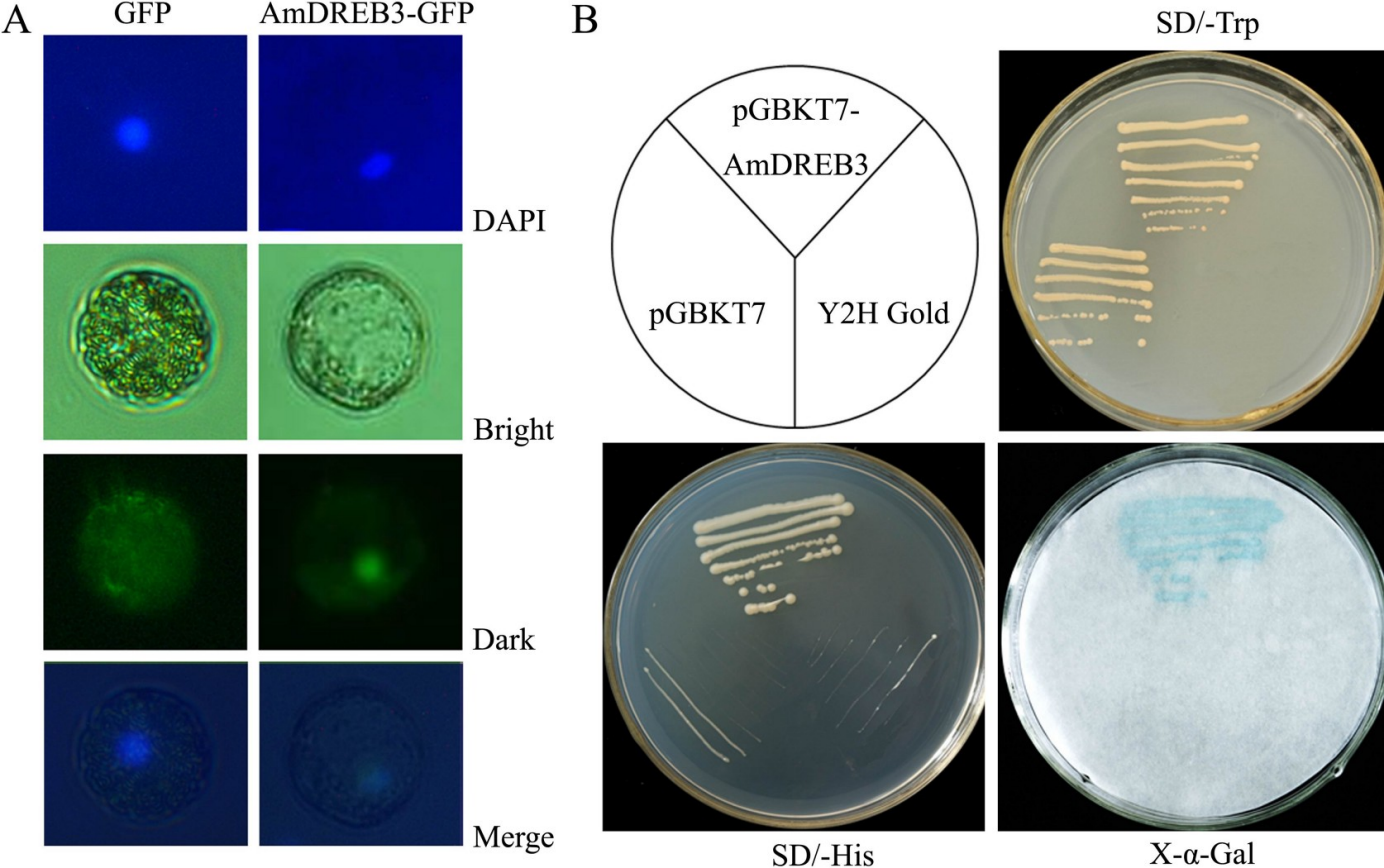
Subcellular localization and transcriptional activation analysis of the AmDREB3 protein. The pBI-AmDREB3:GFP fusion structure and control plasmid pBI-GFP were separately transferred into Arabidopsis leaf protoplasts using a polyethylene glycol-mediated method. (A) The images under dark, bright, and merged fields are presented. (B) Growth of the transformants with pGBKT7-AmDREB3, pGBKT7, or Y2HGold (the latter two were used as negative controls) on SD/-Trp or SD/^-^His medium and the blue color in the X-α-Gal assay are shown.

To provide evidence for the potential role of AmDREB3 in transcriptional regulation, we first examined the subcellular localization of AmDREB3-GFP in Arabidopsis protoplasts. As shown in Fig 2A, the signal of AmDREB3-GFP was exclusively located in the nuclei of protoplasts, while that of the control GFP was detected throughout the cells. We next analyzed the transactivation ability of AmDREB3 by introducing the GAL4 DNA-binding domain-AmDREB3 recombinant plasmid into yeast cells and testing the ability to activate transcription of the dual reporter genes *His3* and *MEL1*. Yeast cells with the fusion plasmids harboring *AmDREB3* grew on SD medium lacking His or Trp and were stained blue with X-α-Gal solution, while the negative controls pGBKT7 and Y2HGold did not (Fig 2B). These observations demonstrated that AmDREB3 is present as a nuclear protein and has transactivation capability.

### The expression patterns of *AmDREB3* under different abiotic stress conditions and in various organs

**Fig 3:**
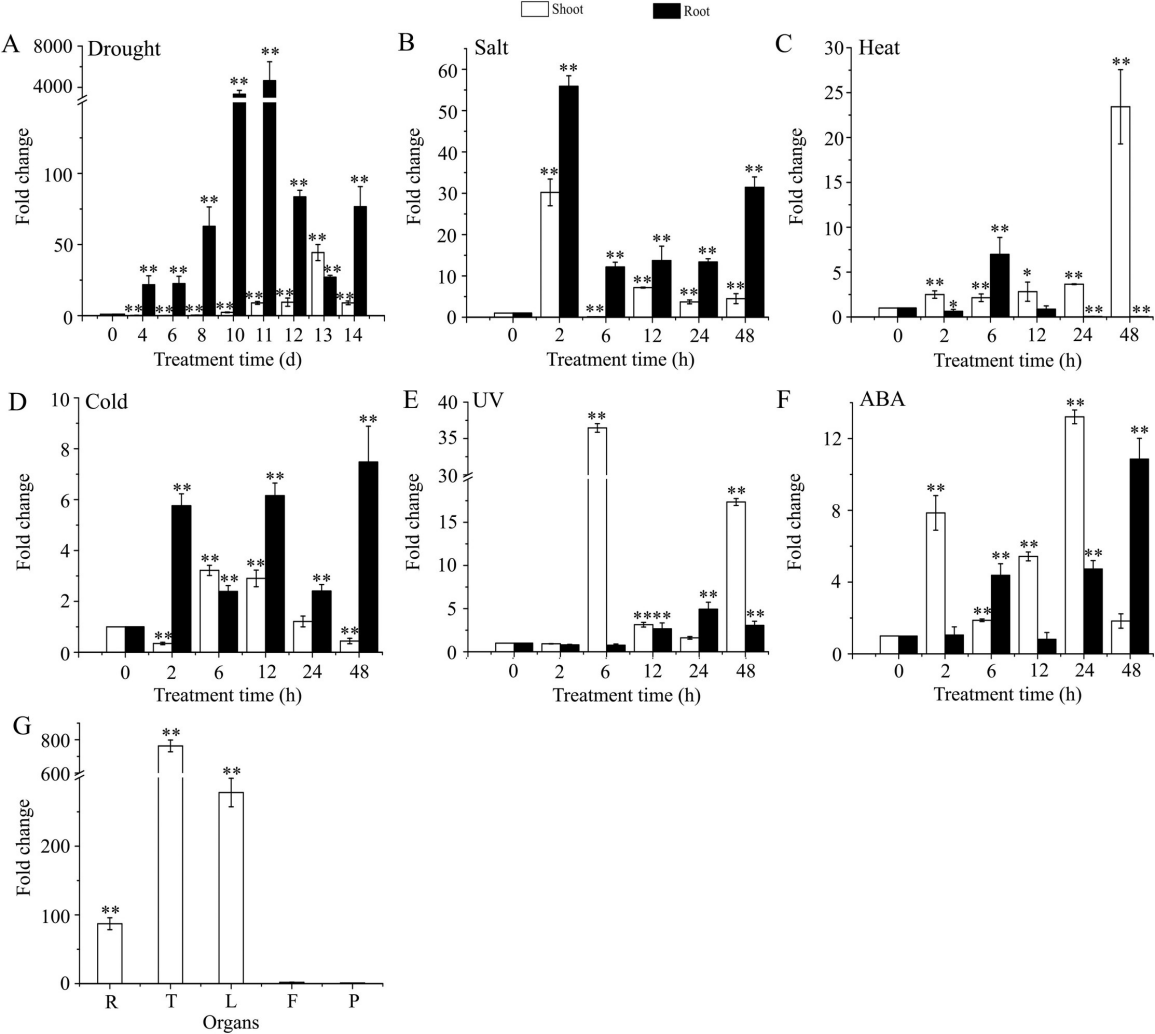
Expression patterns of *AmDREB3* under different abiotic stress conditions and in various organs. One-month-old seedlings were subjected to different abiotic stressors or ABA treatment, and the expression changes of *AmDREB3* in the shoots and roots are presented. (A) The expression changes during the 14 d drought treatment. The transcript level at 0 d was used as the control. (B) to (F) The expression changes during the salt (watering the seedlings with 350 mM NaCl solution), heat (42°C), cold (4°C for 24 h, 0°C for 12 h, and -6°C for 12 h, in total 48 h), UV-B (40 w), and ABA (100 μM) treatments for 2 to 48 h, respectively. (G) The expression pattern in different organs of naturally growing *A*. *mongolicus* shrubs in the wild. R, T, L, F, and P represent roots, twigs, leaves, flower buds, and immature pods, respectively. The α-tubulin gene of *A*. *mongolicus* was used as an internal reference gene. Data are presented as means ± SDs (n = 3). Asterisks and double asterisks indicate significant differences between each stress treatment or each organ and its control at p < 0.05 and <0.01, respectively.

To further reveal the potential function of *AmDREB3* in response to different stressors and during growth process, we performed expression analysis using RT-qPCR. Under drought treatment, the transcription of *AmDREB3* in roots of laboratory-cultured *A*. *mongolicus* seedlings showed an increasing trend during the 0 to 14 d treatment period, and the transcript level reached the maximum (4638.6-fold higher than the control level at 0 d) at 11 d and declined to the minimum (6.2-fold higher than that at 0 d) at 13 d of the treatment (Fig 3A). However, in the shoots the transcript levels decreased between 4 to 6 d and then started to rise after 8 d and reached the maximum (44.3-fold higher than that at 0 d) on the 13th day after the treatment. Under salt treatment, the transcription in the roots and shoots showed a similar undulate increasing trend with an overall greater magnitude of induced expression in roots compared to that in shoots (Fig 3B). During heat treatment, the transcript levels in the shoots gradually increased and reached the highest level of 23.4-fold by 48 h, while those in the roots showed decreased changes except at 6 h when the level was 6.9-fold higher than that at 0 h (Fig 3C). After exposure to cold treatment, transcription in the shoots decreased rapidly after 2 h and then increased at 6 h (3.2-fold higher than that at 0 h) and maintained the high level up to 12 h before dropping again after 24 h of treatment. Transcription in the roots showed an undulate increasing pattern and peaked at 48 h (7.5-fold higher than that at 0 h) (Fig 3D). *AmDREB3* also exhibited higher inducible transcription in the shoots compared to the roots during the UV-B treatment. The maximum levels reached 36.5-fold (at 6 h) and 4.9-fold (at 24 h) higher than those at 0 h in the shoots and roots, respectively (Fig 3E). The ABA treatment caused differential increase in the transcript levels in both shoots and roots, and the change was more rapid and obvious in the shoots (peaked at 24 h and 13.2-fold higher than that at 0 h) compared to that in the roots (peaked at 48 h and 10.9-fold higher than that at 0 h) (Fig 3F). Collectively, the results indicate that *AmDREB3* was mainly induced in roots under drought and salt stresses, while in shoots it was mainly induced by the heat, UV-B, and ABA treatments.

With respect to the transcription of *AmDREB3* in different organs of naturally growing *A*. *mongolicus* shrubs in the wild, the lowest transcript level was observed in immature pods (used as control), followed by flower buds. In contrast, a higher transcript level was detected in twigs (769.9-fold higher than that of the control), followed by young leaves and lateral roots (Fig 3G), indicating that *AmDREB3* might participate in the transcriptional regulation of genes in these organs.

### Constitutive expression of *AmDREB3* enhanced drought tolerance, anthocyanin accumulation, and oxidative stress tolerance of transgenic Arabidopsis

To investigate the biological function of *AmDREB3* in plants, we constitutively expressed *AmDREB3* in Arabidopsis to generate transgenic plants. Four independent transgenic lines (OE-1, OE-4, OE-13, and OE-15) with moderate or high transcript levels of *AmDREB3* were identified by semi-quantitative RT-PCR analysis after PPT screening and PCR confirmation (S2 Fig). None of these lines showed obvious growth retardation during their whole life cycles under normal growth conditions and were used for further analyses.

#### Improvement of seed germination, seedling growth, and water-holding capacity under drought stress

**Fig 4:**
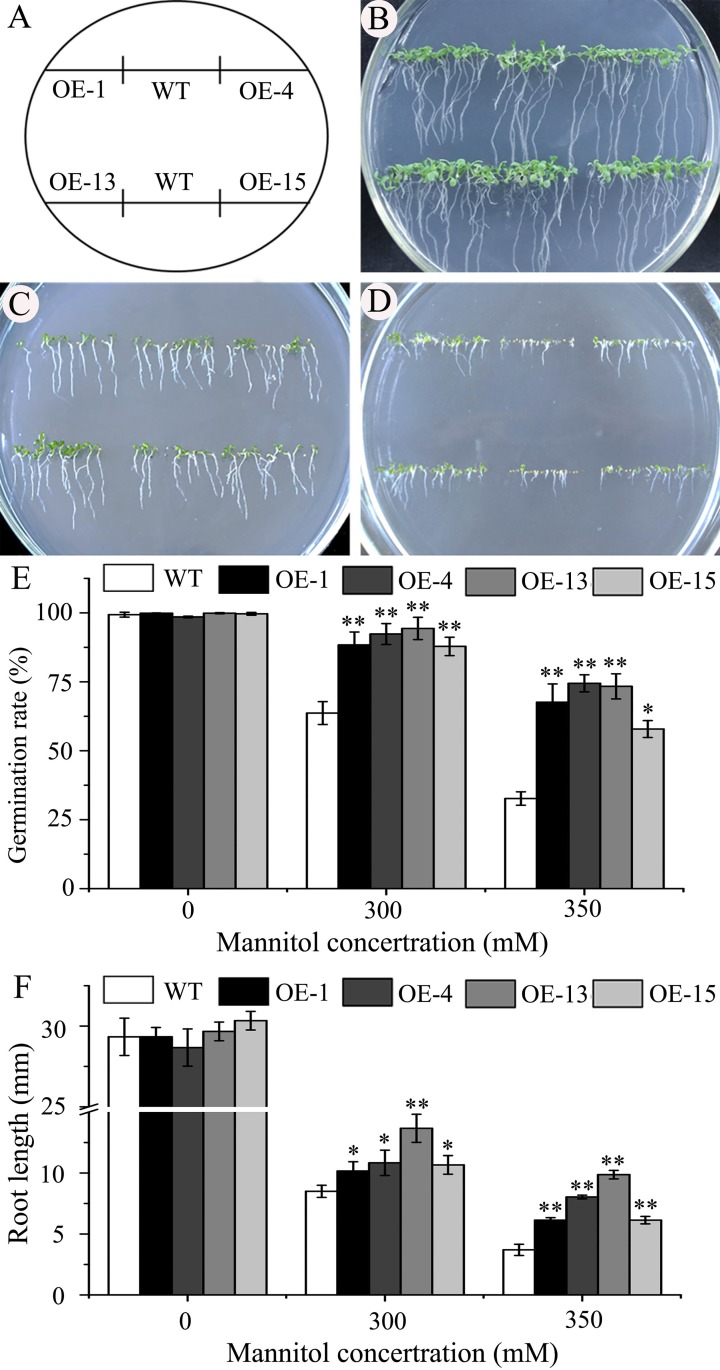
Constitutive expression of *AmDREB3* enhanced osmotic stress tolerance of transgenic Arabidopsis at the germination stage. (A) The location of transgenic lines and wild-type (WT) Arabidopsis on different medium plates. (B to D) After 10 d of germination on only 1/2 MS (0) and 1/2 MS plus 300 or 350 mM mannitol plates, respectively. (E and F) Germination rates and root lengths of seedlings on different media for 3 d and 10 d, respectively. Data are presented as means ± SDs (n = 3). Asterisks and double asterisks indicate significant differences of transgenic lines (OE-1, OE-4, OE-13, and OE-15) compared to wild-type (WT) at p < 0.05 and <0.01 levels, respectively.

Drought tolerance assays at the seed germination stage were performed on 1/2 MS media with 300 or 350 mM mannitol. No significant differences between transgenic lines and wild type were found on 1/2 MS agar plates (Fig 4B). However, both germination and subsequent seedling growth of the transgenic lines were significantly better than those of wild type on the 1/2 MS media containing mannitol, especially with 350 mM mannitol (Fig 4C and 4D). After incubation for 3 d on the medium with 350 mM mannitol, the germination rates of the four lines OE-1, OE-4, OE-13, and OE-15 were 57.9 ± 3.1% to 74.4 ± 4.5%, while that of the wild type was 32.7 ± 2.4% (Fig 4E). After 10 d of germination on the medium with 350 mM mannitol, the root lengths of the transgenic seedlings ranged from 6.1 ± 0.3 to 9.9± 0.3 mm, while that of the wild-type seedlings was 3.7 ± 0.4 mm (Fig 4F).

**Fig 5:**
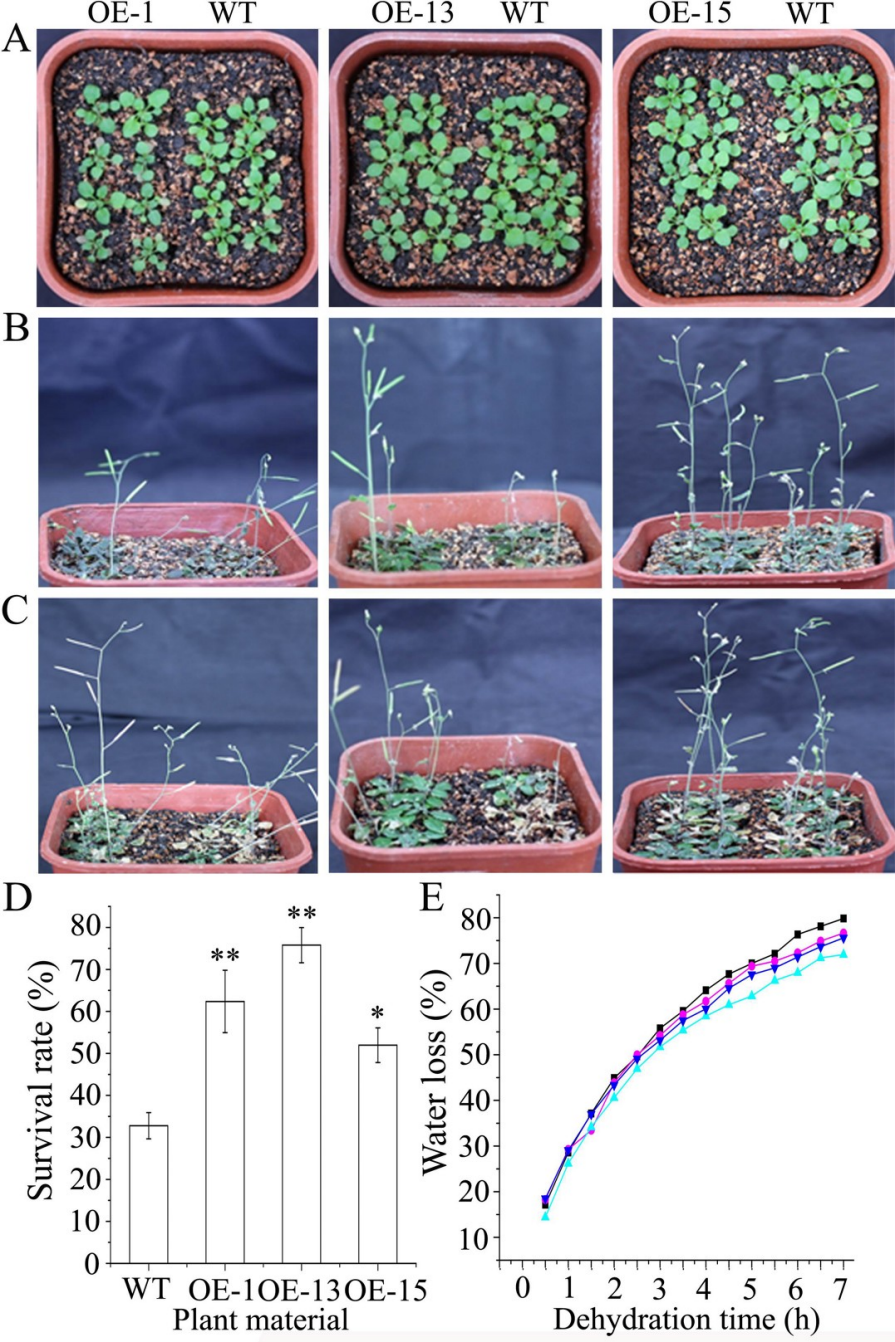
Constitutive expression of *AmDREB3* enhanced drought tolerance of transgenic Arabidopsis at the seedling stage. Ten-day-old seedlings were unwatered for 17 d and then re-watered. (A and B) Seedlings and plants before and 17 d after the suspended watering, respectively. (C and D) Plant phenotypes and survival rates after 7 d of re-watering. (E) Water loss of the detached shoots during a 7-h period. Shoots were weighed at 0.5 h intervals. Water loss is represented as the percentage of initial fresh weight (0 h) at each time point. Data are presented as means. Asterisk and double asterisks indicate significant differences of transgenic lines (OE-1, OE-13, and OE-15) compared to wild-type (WT) at p < 0.05 and <0.01, respectively.

The drought tolerance experiments at the seedling stage were conducted by withholding watering for 17 d and then returning the seedlings to their regular watering schedule. After about 10 d of the drought treatment, the leaf color of the transgenic seedlings turned purple but wild-type seedlings appeared normal except for some slight purple coloration of leaves. After an additional 7 d of the treatment, the wild-type plants became severely wilted, while the transgenic plants showed slight or moderate wilting (Fig 5B). During the 7 d period of re-watering, the transgenic plants recovered quickly, whereas most of the wild-type plants did not recover or even died (Fig 5C). At 7 d after re-watering, the survival rates of the lines OE-1, OE-13, and OE-15 were 51.9 ± 4.1%, 62.4 ± 7.4%, and 75.8 ± 4.2%, respectively, while that of the wild type was 32.8 ± 3.1% (Fig 5D).

The measurement of water loss of detached shoots, an important indicator of plant response to water deficit or drought stressor, showed that the transgenic lines had obviously lower water loss compared to wild-type seedlings after 3.5 h of the dehydration treatment. For instance, at 6 h of treatment the water loss rates of transgenic seedlings were 70.0 to 72.3%, while that of wild-type seedlings was 76.3% (Fig 5E). Together, these results indicate that constitutive expression of *AmDREB3* significantly improved the drought tolerance of transgenic Arabidopsis at the seed germination and seedling stages.

#### Increase in anthocyanin content and drought-induced oxidative stress tolerance

**Fig 6:**
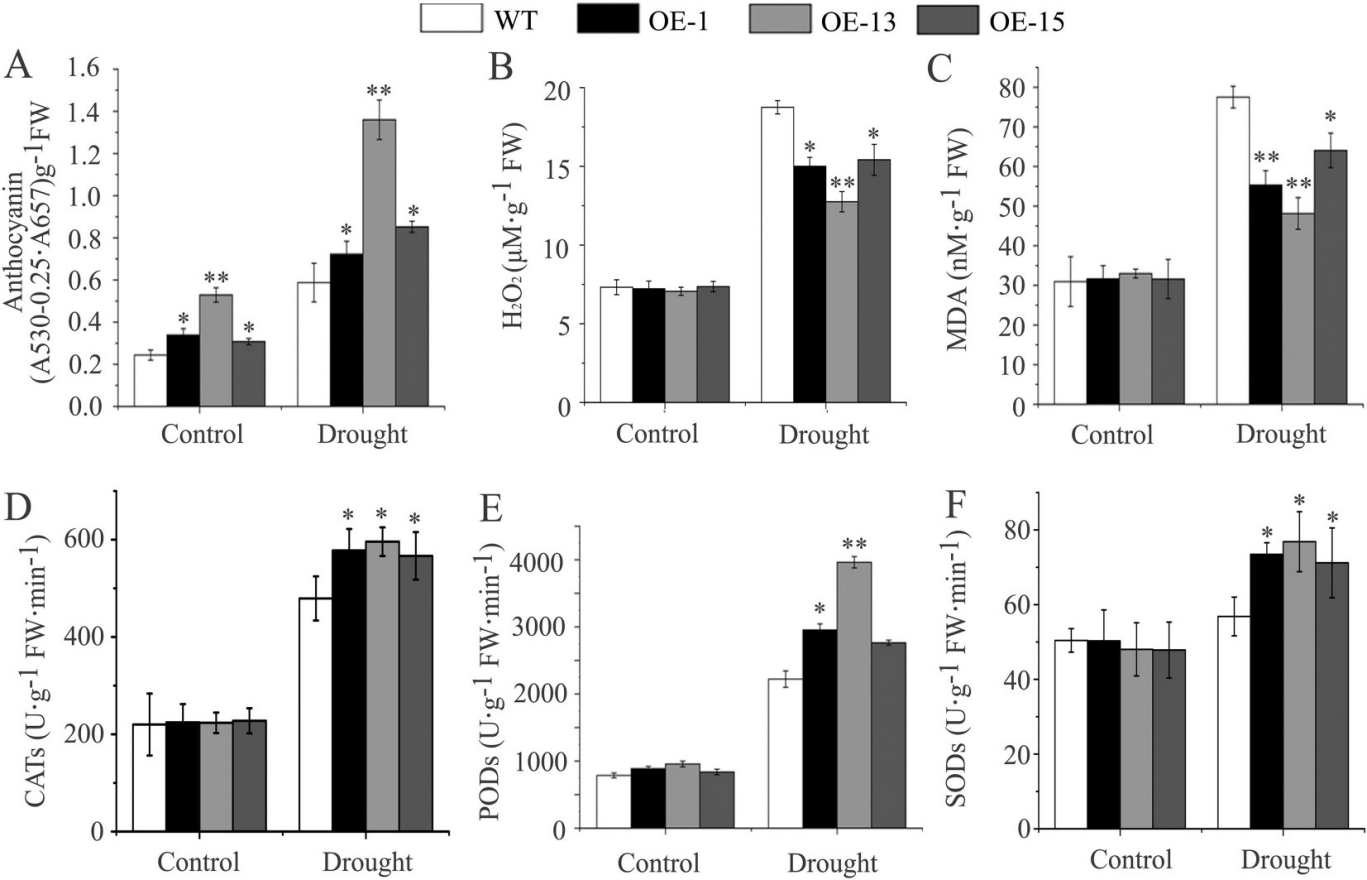
The changes in physiological indices in *AmDREB3* transgenic Arabidopsis under drought stress. Three-week-old seedlings were suspended watering for 10 d, and the contents of anthocyanins, H_2_O_2_, and MDA as well as the activities of CAT, POD, and SOD were measured before (control) and after the drought treatment, respectively. Data are presented as means ± SDs (n = 3). Asterisks and double asterisks indicate significant differences of transgenic lines (OE-1, OE-13, and OE-15) compared to wild-type (WT) at p < 0.05 and <0.01, respectively.

An interesting phenomenon during drought treatment was purple coloration of the leaves of transgenic seedlings (Fig 5). In fact, the leaves of transgenic lines appeared slightly blue-green under normal growth conditions, while leaves of wild type were the expected green. Therefore, we examined changes in anthocyanins, a well-known index for purple coloration of leaves, under drought treatment. The contents of anthocyanins in transgenic seedlings were greater than that in wild-type seedlings in normal growth conditions. After exposure to the drought treatment for 10 d, the content increased in both sets of plants. However, the increase was greater in the transgenic lines compared to the wild-type. At the end of drought treatment, the anthocyanin contents of three transgenic lines increased, on average, by 115.6%, whereas the wild type increased by 100.0% (Fig 6A).

Anthocyanins play an important role in plant tolerance to oxidative stress caused by the overproduction of ROS under abiotic stresses. Therefore, we measured the content of H_2_O_2_, an important class of ROS, and MDA, a major product of lipid peroxidation (LPO) triggered by excessive ROS, after drought treatment. As shown in Fig 6B and 6C, there were no obvious differences in these indices between the transgenic lines OE-1, OE-13, and OE-15 and their wild-type controls under normal conditions. After exposure to drought treatment, however, significantly smaller increases in both H_2_O_2_ and MDA contents were observed in all three transgenic lines (average increases of 93.0% and 70.5%, respectively) compared to those of wild type (respectively increased by 156.1% and 150.4%). Moreover, the activities of CATs, PODs, and SODs, the important antioxidant enzymes, showed significantly greater increases in the transgenic lines (average increases of 157.6%, 258.8%, and 51.5%, respectively) compared to those of the wild type (increased by 117.8%, 182.4%, and 12.7%, respectively) (Fig 6D, 6E and 6F).

All these data indicate that the transgenic lines had an increased capacity to accumulate anthocyanins and concomitantly improve tolerance to drought-induced oxidative stress.

### Constitutive expression of *AmDREB3* enhanced salt tolerance, anthocyanin accumulation, and oxidative stress tolerance of transgenic Arabidopsis

#### Alteration in phenotype and survival under salt stress

**Fig 7:**
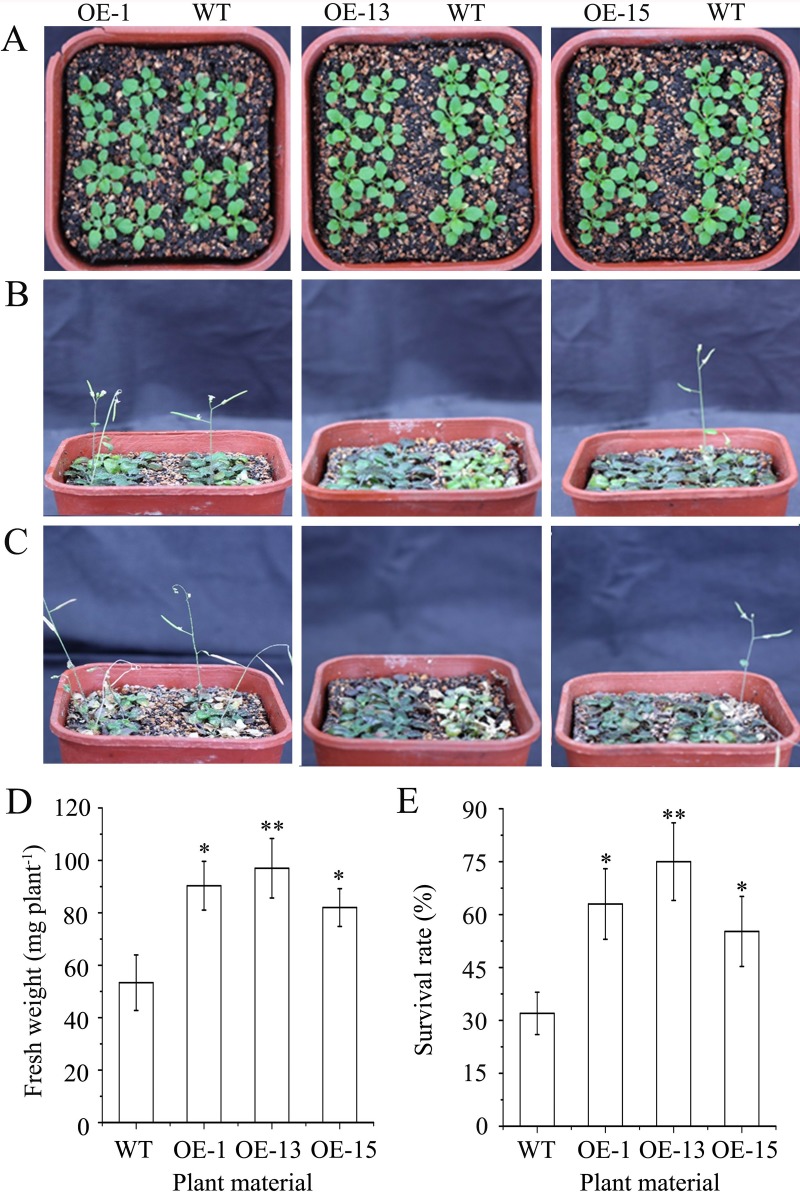
Constitutive expression of *AmDREB3* enhanced salt tolerance of transgenic Arabidopsis at the seedling stage. Ten-day-old seedlings were watered with a 300 mM NaCl solution once and then were returned to their regular watering schedule of once every 5 d with tap water. (A, B and C) Plant phenotypes of the transgenic lines (OE-1, OE-13, and OE-15) and wild-type (WT) Arabidopsis before, 7 d after, and 14 d after watering with the NaCl solution, respectively. (D and E) Fresh weights and survival rates of plants after 7 d and 14 d of watering with the NaCl solution, respectively. Data are presented as means ± SDs (n = 3). Asterisks and double asterisks indicate significant differences of transgenic lines (OE-1, OE-13, and OE-15) compared to wild-type (WT) at p < 0.05 and <0.01, respectively.

Salt stress was imposed by watering seedlings with a 300 mM NaCl solution. Like the earlier drought treatment, most leaves of the transgenic seedlings turned purple after 3 to 4 d of watering with the NaCl solution. In contrast, only some leaves of the wild-type seedlings became slightly purple in the same period. During the next two to three weeks when the seedlings were returned to their regular watering schedule, most of the transgenics became more purple in leaf color with some bleaching of leaf margins, while the wild type turned chlorotic, had bleaching of most leaves, or even died (Fig 7C). After one week of watering with the NaCl solution, the fresh weights of all of the tested transgenic seedlings were significantly greater than that of the wild-type seedlings (Fig 7D). When measured after three weeks of watering with the NaCl solution, survival rate of the wild-type plants was 32.5 ± 6.0%, whereas those of the OE-1, OE-13, and OE-15 lines were 63.2 ± 13.3%, 75.4 ± 13.2%, and 55.2 ± 9.9%, respectively (Fig 7E). These results show that *AmDREB3* plays an important role in improving salt tolerance, which may closely correlate with the increase in anthocyanin synthesis.

#### Increase in anthocyanin content and salt-induced oxidative stress tolerance

**Fig 8:**
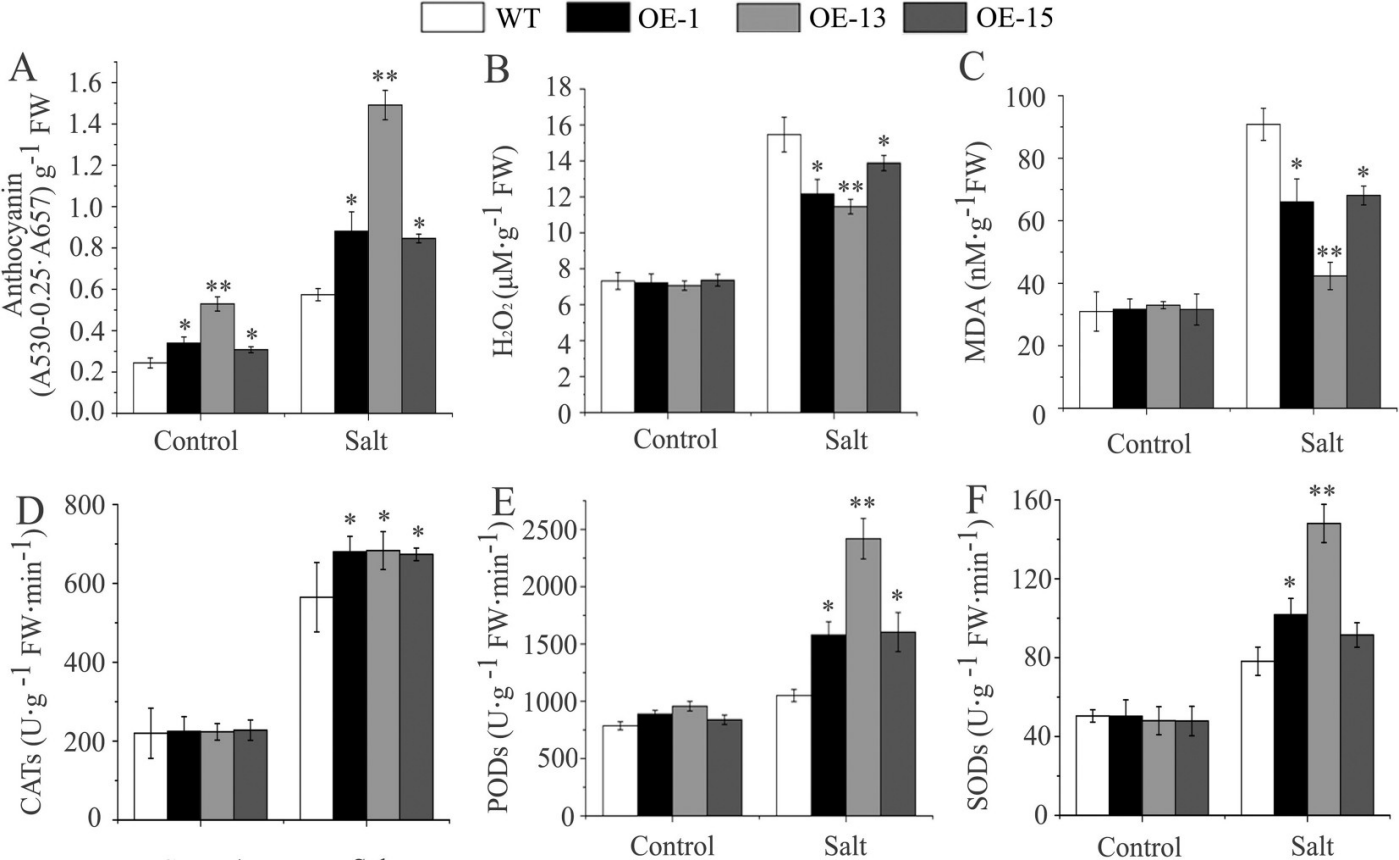
The changes in physiological indices in *AmDREB3* transgenic Arabidopsis under salt stress. Three-week-old seedlings were watered with a 300 mM NaCl solution, and the contents of anthocyanins, H_2_O_2_, and MDA as well as the activities of CAT, POD, and SOD were measured before (control) and 5 d after watering with the NaCl solution. Data are presented as means ± SDs (n = 3). Asterisks and double asterisks indicate significant differences of transgenic lines (OE-1, OE-13, and OE-15) compared to wild-type (WT) at p < 0.05 and <0.01, respectively.

As shown in Fig 8A, the contents of anthocyanins in the transgenic lines OE-1, OE-13, and OE-15 were 1.4, 2.2, and 1.3 times that in the wild-type seedlings, respectively, under normal conditions. After 5 d of watering with a 300 mM NaCl solution, the anthocyanin contents in all transgenic lines (OE-1, OE-13, and OE-15) increased by 159.6%, 181.9%, and 174.8%, whereas that in the wild-type seedlings increased by only 135.7%. By comparison, the magnitude of the salt-induced anthocyanin accumulation in all three transgenic lines was greater than that under the drought stress described earlier (Fig 6A).

Like the drought treatment described above, the increase in both H_2_O_2_ and MDA contents in transgenic lines was obviously smaller than that in wild type after the salt treatment. However, the increases in the CAT, POD, and SOD activities were obviously greater in the transgenic lines than in the wild type (Fig 8B, 8C, 8D, 8E and 8F).

These results demonstrate that constitutive expression of *AmDREB3* enhanced anthocyanin accumulation and oxidative stress tolerance under salt stress.

### Constitutive expression of *AmDREB3* enhanced heat tolerance and anthocyanin accumulation of transgenic Arabidopsis

**Fig 9:**
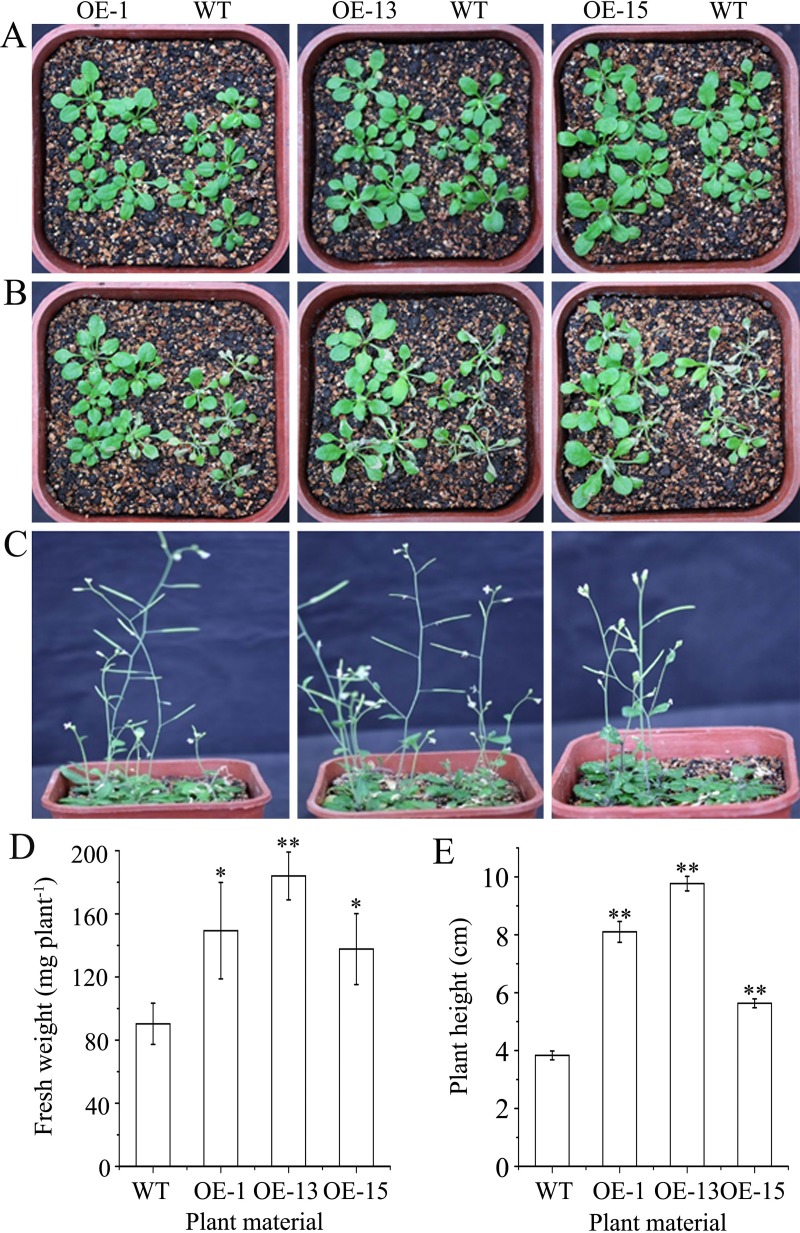
Constitutive expression of *AmDREB3* enhanced heat stress tolerance of transgenic Arabidopsis. Ten-day-old seedlings were placed at 53°C for 2 h after a pretreatment at 37°C for 1 h and at 24°C for an additional 2 h. Then the seedlings were returned to normal conditions for recovery. (A) Seedlings of transgenic lines (OE-1, OE-13, and OE-15) and wild-type (WT) Arabidopsis before the heat treatment. (B and C) Phenotypes of the plants at 3 d and 14 d after the heat treatment, respectively. (D and E) Fresh weights and plant heights after 14 d of recovery, respectively. Data are presented as means ± SDs (n = 3). Asterisks and double asterisks indicate significant differences of transgenic lines compared to WT at p < 0.05 and <0.01, respectively.

Heat tolerance was tested by placing seedlings at 37°C for the first 1 h, at 24°C for the next 2 h, and at 53°C for the last 2 h. At the end of the treatment, leaves of most of the wild-type seedlings were obviously withered, while the transgenic seedlings showed slight withering of some leaves. After a few days of recovery under normal growth conditions, most of the transgenic seedlings continued to grow well, whereas resumed growth in the wild-type seedlings was poor. After two weeks of recovery, the fresh weights and plant heights of the OE-1, OE-13, and OE-15 lines ranged from 137.7 ± 22.5 to 184.0 ± 15.1 mg·plant^-1^ and from 5.6 ± 0.2 to 9.8 ± 0.3 cm, respectively, while those of the wild type were respectively 90.3 ± 13.1 mg·plant^-1^ and 3.8 ± 0.2 cm (Fig 9D and 9E). Moreover, an obvious purple coloration of the main veins and petioles of rosette leaves and the basal stems of the transgenic plants, but not the wild-type plants, were observed during the recovery period (Fig 9C). These results indicate that *AmDREB3* expression enhanced heat shock tolerance and anthocyanin accumulation in the heat-stressed parts of transgenic Arabidopsis.

We also evaluated the effect of *AmDREB3* expression on the tolerance to cold and exogenous ABA treatments, but no obvious differences were observed between the transgenic lines and wild type (S3 Fig and S4 Fig).

### Constitutive expression of *AmDREB3* induced the transcription of stress-inducible genes in transgenic Arabidopsis

**Fig 10:**
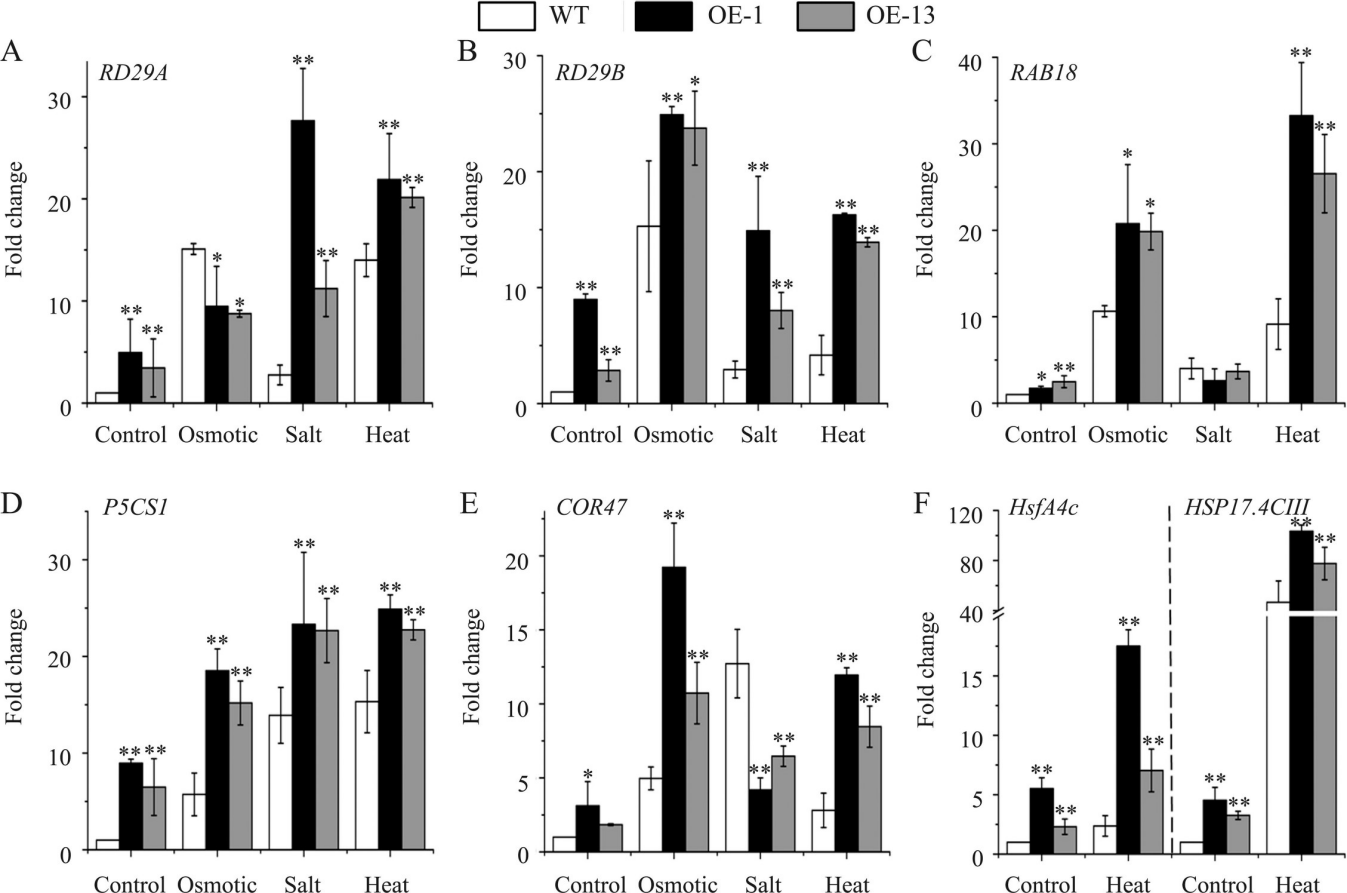
Transcript levels of seven stress-inducible genes in *AmDREB3* transgenic Arabidopsis. The transcript levels of seven stress-inducible genes in two-week-old seedlings were examined by RT-qPCR analysis. The seedlings were exposed to osmotic stress (350 mM mannitol), salt stress (175 mM NaCl), or heat stress (37°C) for 5 h before sampling. Asterisks and double asterisks indicate significant differences of transgenic lines (OE-1 and OE-13) compared to wild-type (WT) at p < 0.05 and <0.01, respectively.

As a class of TFs, DREBs improve stress tolerance by regulating the transcription of many stress-inducible genes [1,3]. In Arabidopsis, the stress-inducible genes *RD29A*, *RD29B*, *RAB18*, *COR47*, and *P5CS1* are commonly used to explore the molecular mechanisms of TFs in the improvement of stress tolerance in transgenic plants. *HsfA4c* and *HSP17*.*4-CⅢ* can also be used as sensitive markers for heat stress response [37]. Here, we found that the transcript levels of all these genes were increased to between 1.2- and 12.0-fold in transgenic lines compared to those in wild type under normal conditions (Fig 10). After exposure to osmotic, salt, or heat stress treatment for 5 h, the transcription of all these genes increased in both sets of plants, and the transgenic lines had still higher transcript levels of almost all of these genes compared to their wild-type controls under these stresses. In particular, the transcript levels of *RD29B*, *RAB18*, *P5CS1*, and *COR47* were higher in the transgenics than in wild type under osmotic stress. However, *RDD29A*, *RD29B*, and *P5CS1* had higher transcript levels in transgenic lines than in wild type under salt stress. After exposure to heat, the transcript levels of all the examined stress-inducible genes were higher in the transgenic lines compared to those in wild type (Fig 10). These results are consistent with the increased tolerance of the transgenic lines to drought, salt, and heat stressors (Figs 4, 5, 7 and 9), suggesting that AmDREB3 promoted the expression of some stress-inducible genes in transgenic Arabidopsis.

### Analysis of *cis*-regulatory elements in the *AmDREB3* promoter region

A 1143 bp promoter sequence (from transcription start site) of *AmDREB3* was isolated from the genomic DNA of *A*. *mongolicus* seedlings. Using the Plant *Cis-*acting Regulatory DNA Element, ten classes of *cis*-acting elements were predicted to be involved in the transcriptional regulation of *AmDREB3* (Table 1). The TATA-box and CAAT-box, two basic transcriptional regulatory elements, were respectively detected at approximately -30 and -78 bp upstream of the transcription start site. Three to 7 (in total 26) recognition sites for the MYB, MYC, DOF, WRKY, ABF/AREB, GT-1, or AAR1 TFs were also detected, most of which participate in abiotic stress response in plants [1,43–44]. In addition, 3 CGCG boxes recognized by AtSRs were found (Table 1). These results imply that the transcription of *AmDREB3* might be co-regulated by multiple upstream regulators.

**Table 1:** Predicted *AmDREB3* promoter *cis*-acting elements.

Name	Sequence	Number	Location	Function
DOFCOREZM	AAAG	6	-140/-209/-508/-720/-816/-1070	Core site required for binding of Dof proteins
MB	GGATA	4	-115/-376/-678/-1083	MYB recognition site
MC	CANNTG	4	-349/-367/-421/-767	MYC recognition site
ABRE	MACGYGB	3	-348/-366/-420	ABA responsive element
ARR1AT	NGATT	3	-756/-1025/-1119	Arabidopsis response-regulator 1 (ARR1) binding element
CGCG-box	VCGCGB	3	-396/-409/-468	‘CGCG box’ recognized by *A*. *thaliana* signal-responsive genes (AtSRs)
GT-1	GRWAAW	3	-135/-314/-500	Consensus GT-1 binding site in many light-regulated genes
W-box	TTGAC	3	-413/-516/-982	WRKY DNA binding proteins recognized specifically site
CCAT-box	CCAAT	1	-78	Common sequence found in the 5'-non-coding regions of eukaryotic genes
TATA-box	TATAAA	1	-30	Common sequence found in the 5'-non-coding regions of eukaryotic genes

## Discussion

### AmDREB3 is a novel member with transcriptional activation activity in the A-5 subgroup of DREB subfamily

DREB TFs are considered master switches that, in response to abiotic stressors, activate the expression of many relevant downstream genes, the so called ‘regulon’ [10,45]. *Ammopiptanthus mongolicus* is emerging as a model species for studying plant tolerance to harsh environments [35–37]. Based on our previous work for *DREBs* in *A*. *mongolicus* [35,37], a novel *DREB* gene, *AmDREB3*, was cloned and functionally analyzed in the present study. *AmDREB3* was classified into the A-5 subgroup of the *DREB* subfamily; the entire sequence of AmDREB3 protein shares the highest homology with soybean GmDREB3 among all of the accepted DREB sequences in GenBank. In the AP2 domain, AmDREB3 has the same conserved 14th V as all other members in the A-5 subgroup but contains a 19th L instead of a conserved 19th E like other A-5 subgroup DREBs, except for GmDREB3 and PeDREB2a that also have a 19th L (Fig 1). This finding provides further evidence that the 14 V might be more important for DREB binding specifically to the DRE in their regulon’s promoters compared with the 19 E [46–47]. In addition, sequence alignments showed that AmDREB3 does not contain the typical EAR-motif (DLNxxP) that exists in most of the other A-5 subgroup DREBs, the same as soybean GmDREB3, *P*. *patens* PpDBF1, *P*. *euphratica* PeDREB2a, and *S*. *caninervis* ScDREB8 (Fig 1). The EAR-motif has been identified as an essential domain in transcriptional repressors of plants [48] and was found to be necessary for transcriptional repression of the EAR-motif-containing GhDBP1 and AtRAP2.1 in the A-5 subgroup [6–7]. Previous studies have demonstrated that the PpDBF1, GmDREB3, PeDREB2a, and ScDREB8 proteins that do not contain the EAR-motif acted as transcriptional activators of reporter genes in yeast cells and/or DRE-mediated genes in transgenic plants [12,14,16,49]. Our results (Fig 2B and Fig 10) are similar to these findings, providing further evidence that the EAR-motif may be crucial to transcriptional repression of the A-5 subgroup DREB TFs. However, some of the EAR-motif-containing DREBs, such as soybean GmDREB1 and GmDREB2 and potato StDREB2 (Fig 1), have been confirmed to activate transcription of the reporter and/or DRE-mediated genes in transgenics [10–11,13]. It is thus clear that differential regulatory mechanisms exist among the A-5 subgroup DREB TFs in response to various abiotic stressors. Therefore, more studies are needed to better understand the actual roles and detailed mechanisms of the A-5 subgroup DREBs in regulating plant response to different stressors.

### *AmDREB3* may play important regulatory roles in plant tolerance to drought, salt, and heat stressors

Previous researches on *DREBs* have focused on the A-1 and A-2 subgroups. The A-1 subgroup members (*DREB1s/CBFs*) are mainly involved in the response to low temperature and the tolerance to cold, drought, and salt stressors; the A-2 subgroup members (*DREB2s*) primarily respond to and tolerate drought, salt, and heat stressors [1,3,5]. Here, *AmDREB3* was found to be significantly induced in the roots and/or shoots of different stress- and ABA-treated *A*. *mongolicus* seedlings (Fig 3). Furthermore, overexpressing *AmDREB3* in Arabidopsis conferred tolerance against drought, salt, and heat stressors (Figs 4, 5, 7 and 9), likely by activating or up-regulating the expression of some stress-inducible genes (Fig 10), but had no obvious effects on cold tolerance and ABA sensitivity (S3 Fig and S4 Fig). Thus, *AmDREB3* is more similar to *DREB2s* rather than *DREB1s/CBFs* [1,3,5]. However, *GmDREB3*, the closest ortholog of *AmDREB3* among all of the *DREBs* in GenBank, was induced only by cold and its overexpression increased tolerance to cold, drought, and high salinity of transgenic plants [12]. Therefore, *GmDREB3* is closer to *DREB1s/CBFs* but not *DREB2s* in both expression and function under abiotic stresses. Among the A-5 subgroup genes identified in different species, *AmDREB3* is quite similar to *GmDREB2*, *PpDBF1*, *PeDREB2a*, *HhDREB2*, and *ScDREB8*. All these genes were drought-, cold-, salt- and/or ABA-inducible and could improve drought and salt tolerances of their transgenic plants [11,14–16,48]. In contrast, *AmDREB3* is distinct from *GhDBP1* and *AtRAP2*.*1*, both which acted as negative regulators of stress-inducible gene expression and stress tolerance formation [6–8].

Consistent with the stress responses described above, at least 14 putative recognition sites for MYB, MYC, ABF/AREB, and WRKY TFs were found in the promoter sequence of *AmDREB3* (Table 1). All the four types of TFs are closely involved in the response of plants to various abiotic stressors [1,43–44]. Therefore, it is reasonable to speculate that *AmDREB3* might be tightly controlled by a sophisticated transcriptional regulatory network to maintain a suitable response to multiple stressors, such as drought, salt, and heat.

Combining our results with previous findings mentioned above, we speculate that *AmDREB3* may play important regulatory roles in plants tolerating drought, salt, and heat stressors.

### *AmDREB3* may play a positive regulatory role in anthocyanin biosynthesis and oxidative stress tolerance

Increased biosynthesis of anthocyanins in vegetative tissues under stress conditions is a hallmark of plants against various stressors [19,31,50–51]. So far, anthocyanin biosynthesis has been shown to be mainly regulated at the transcriptional level by the members of the MYB, bHLH, WD40, and NAC TF families as well as the DREB subfamily. All these TFs can positively or negatively regulate anthocyanin biosynthesis under certain conditions or at specific developmental stages of plants [29–34]. In the present study, an obvious purple pigmentation of seedlings was observed in the *AmDREB3* transgenic lines under stress conditions, especially after salt treatment (Figs 5, 7 and 9), caused by high accumulation of anthocyanins (Figs 6 and 8). Even in normal growth conditions, the transgenic seedlings also showed greater anthocyanin contents than their wild-type control (Figs 6 and 8). These observations suggest that AmDREB3 may play a positive regulatory role in the biosynthesis of anthocyanins. Similar phenomena have been observed in the transgenics of a few A-1 subgroup members, including peach *PpCBF1*, eucalyptus *EguCBF1s*, and rice *RdreB1BI*/*OsDREB1B*. All these genes led to a parallel increase in anthocyanin contents and freezing tolerance of their transgenic plants [30–32,34]. However, our observations differ from the findings of Pino et al. [29] and An et al. [33] in that both Arabidopsis *AtCBF1* and *AtCBF3* (both belong to the A-1 subgroup) reduced anthocyanin contents although they enhanced the tolerance of transgenic plants to cold and/or drought stress. Our data provide first evidence of the A-5 subgroup DREBs involved in anthocyanin biosynthesis and new support for functional divergence of *DREB* genes during speciation or plant adaptation to different environments.

Overproduction of ROS is another feature of plant cells during different stresses, which leads to oxidative stress and thus results in more severe injury to plants [52–53]. A major physiological function of anthocyanins is to protect plants from stress-induced oxidative damage by scavenging excessive ROS [54]. H_2_O_2_ is a major and stable ROS. At high levels, it can cause oxidative damage to plant cells [52,55]. Lipid peroxidation is one of the well-known damaging processes to occur under various stresses and is usually estimated by measuring the accumulation of MDA, a peroxidation product of membrane lipids in plant cells. Malondialdehyde can also cause cellular damage by reacting with proteins, other lipids, and nucleic acids [52]. Plant cells are equipped with a set of antioxidant enzymes such as CATs, PODs, and SODs to scavenge ROS. In this study, significantly lower H_2_O_2_ and MDA contents and higher CAT, POD, and SOD activities were detected in *AmDREB3* transgenic lines than in wild-type seedlings after drought and salt treatments (Figs 6 and 8). These results indicate that increased anthocyanin accumulation and antioxidant enzyme activity may play crucial roles in the alleviation of stress-triggered oxidative damage to transgenic plants. *Ammopiptanthus mongolicus* is naturally distributed in the central Asian desert and is generally exposed to harsh environments [35–37]. Here, we detected a universal increase in *AmDREB3* transcript level in the drought-, salt-, heat-, cold-, and UV-treated *A*. *mongolicus* seedlings (Fig 3). It is likely that *AmDREB3* might also function in protecting wild-grown *A*. *mongolicus* shrubs against the oxidative damage triggered by harsh environments and thus contribute to the shrub’s high stress tolerance.

## Conclusions

In this paper, we identified a novel A-5 subgroup DREB gene named *AmDREB3* from *A*. *mongolicus* with high tolerance to harsh desert environments. Its encoding protein was located in the nucleus and has transcriptional activation capacity. The transcription of *AmDREB3* was mainly induced in roots under drought and salt stresses, while in shoots it was mainly induced by heat, UV-B, and ABA treatments. Constitutively expressing *AmDREB3* in Arabidopsis improved tolerance to drought, salt, and heat stressors, which may have been achieved by up-regulating the expression of some important stress-inducible genes. The expression of *AmDREB3* also enhanced anthocyanin accumulation and/or oxidative stress tolerance of transgenics under drought, salt, and heat stresses, which may constitute an important physiological foundation for stress tolerance of transgenic plants. This work provides evidence of the potential effects of *AmDREB3* on *A*. *mongolicus*’ endurance to harsh environments and adds new insight into the mechanisms of how A-5 subgroup DREB TFs function in stress tolerance in plants.

## Supporting information

S1 Fig Phylogenetic tree of AmDREB3 and other DREBs from different plant species. (TIF)

S2 Fig Expression of *AmDREB3* in T_2_ transgenic Arabidopsis lines. (TIF)

S3 Fig The germination phenotype of *AmDREB3* constitutive expression lines under mannitol stress treatment. (TIF)

S4 Fig Evaluation of freezing tolerance of *AmDREB3* transgenic Arabidopsis lines at the seedling stage. (TIF)

S5 Fig Evaluation of ABA sensitivity of *AmDREB3* transgenic Arabidopsis lines at the seed germination stage. (TIF)

S1 Table List of primers used in this study. (DOCX)

S2 Table The DREB proteins used in the construction of the phylogenetic tree and their accession numbers. (DOCX)

S3 Table The sequence of *AmDREB3* promoter region. (DOCX)
