# Correction: Constitutive expression of an A-5 subgroup member in the DREB transcription factor subfamily from *Ammopiptanthus mongolicus* enhanced abiotic stress tolerance and anthocyanin accumulation in transgenic Arabidopsis

**DOI:** 10.1371/journal.pone.0227290

**Published:** 2019-12-26

**Authors:** Meiyan Ren, Zhilin Wang, Min Xue, Xuefeng Wang, Feng Zhang, Yu Zhang, Wenjun Zhang, Maoyan Wang

Fig 5 is missing symbol legends. Please see the correct Fig 5 here.

**Fig 5:**
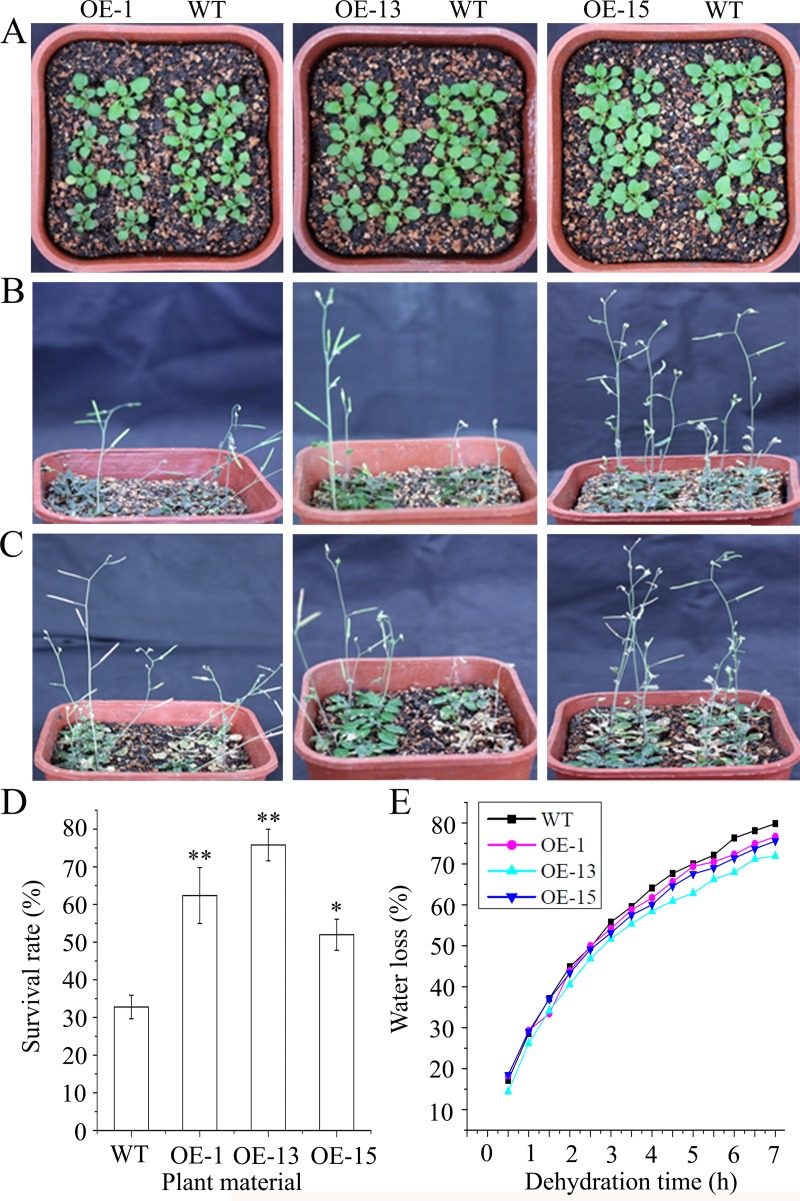
Constitutive expression of *AmDREB3* enhanced drought tolerance of transgenic Arabidopsis at the seedling stage. Ten-day-old seedlings were unwatered for 17 d and then re-watered. (A and B) Seedlings and plants before and 17 d after the suspended watering, respectively. (C and D) Plant phenotypes and survival rates after 7 d of re-watering. (E) Water loss of the detached shoots during a 7-h period. Shoots were weighed at 0.5 h intervals. Water loss is represented as the percentage of initial fresh weight (0 h) at each time point. Data are presented as means. Asterisk and double asterisks indicate significant differences of transgenic lines (OE-1, OE-13, and OE-15) compared to wild-type (WT) at p < 0.05 and < 0.01, respectively.

Fig 7 shows an incorrect image of transgenic line OE-15. Please see the correct Fig 7 here.

**Fig 7:**
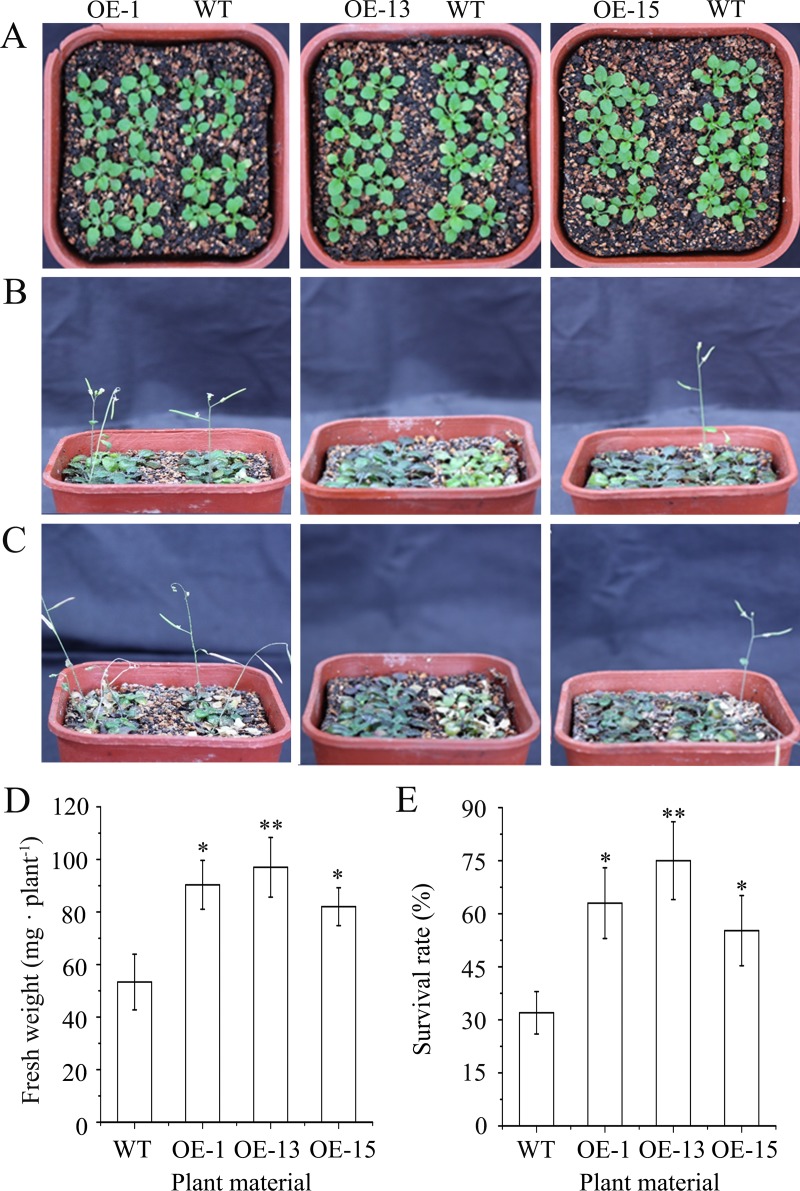
Constitutive expression of *AmDREB3* enhanced salt tolerance of transgenic Arabidopsis at the seedling stage. Ten-day-old seedlings were watered with a 300 mM NaCl solution once and then were returned to their regular watering schedule of once every 5 d with tap water. (A, B and C) Plant phenotypes of the transgenic lines (OE-1, OE13, and OE-15) and wild-type (WT) Arabidopsis before, 7 d after, and 14 d after watering with the NaCl solution, respectively. (D and E) Fresh weights and survival rates of plants after 7 d and 14 d of watering with the NaCl solution, respectively. Data are presented as means ± SDs (n = 3). Asterisks and double asterisks indicate significant differences of transgenic lines (OE-1, OE-13, and OE-15) compared to wild-type (WT) at p < 0.05 and < 0.01, respectively.

Fig 8 is missing the abscissa names “Control” and “Salt.” Please see the correct Fig 8 here.

**Fig 8:**
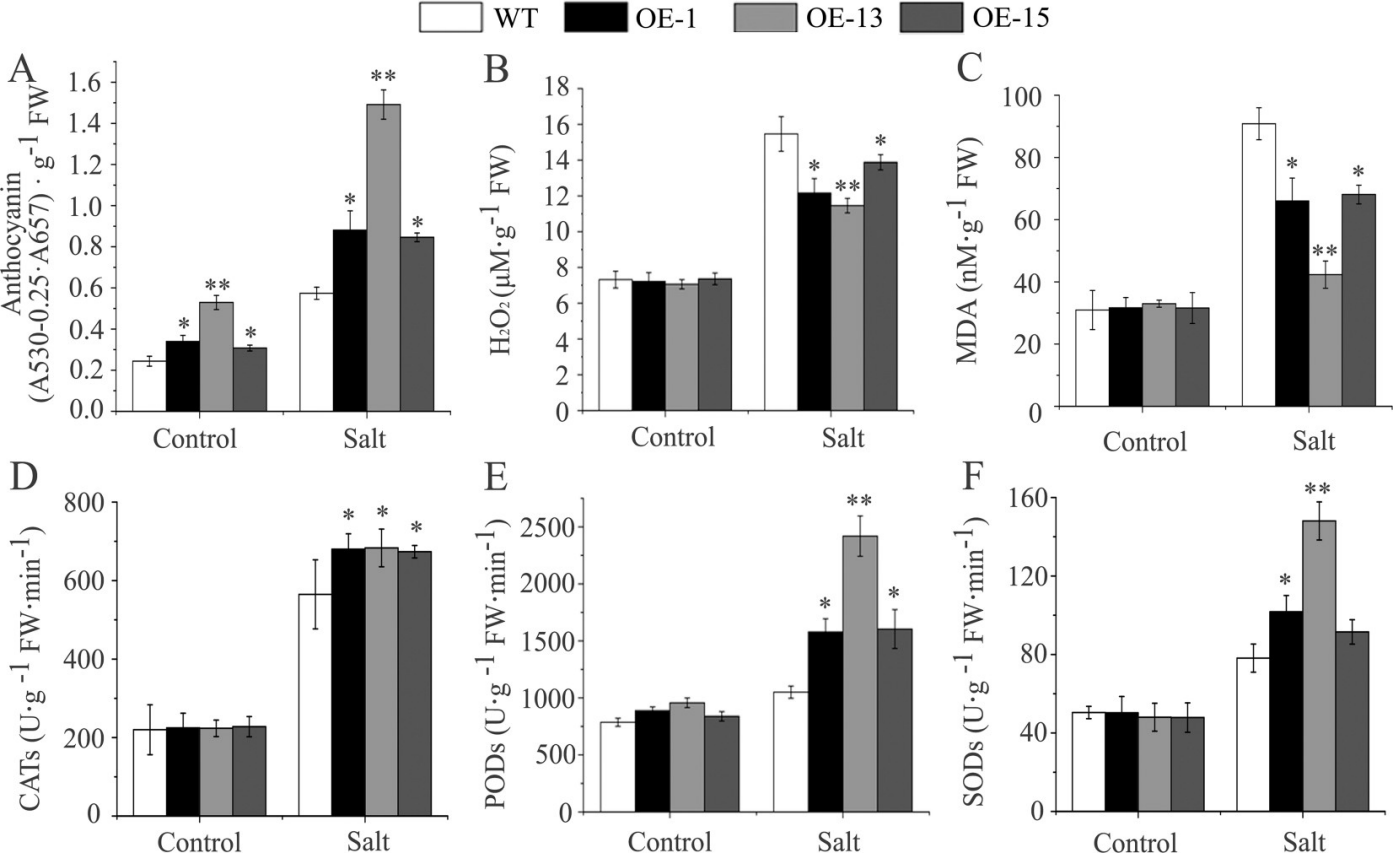
The changes in physiological indices in *AmDREB3* transgenic Arabidopsis under salt stress. Three-week-old seedlings were watered with a 300 mM NaCl solution, and the contents of anthocyanins, H2O2, and MDA as well as the activities of CAT, POD, and SOD were measured before (control) and 5 d after watering with the NaCl solution. Data are presented as means ± SDs (n = 3). Asterisks and double asterisks indicate significant differences of transgenic lines (OE-1, OE-13, and OE-15) compared to wild-type (WT) at p < 0.05 and < 0.01, respectively.

The last sentence of the Constitutive expression of *AmDREB3* enhanced heat tolerance and anthocyanin accumulation of transgenic Arabidopsis subsection of the Results is incorrect. The correct sentence is: We also evaluated the effect of AmDREB3 expression on the tolerance to cold and exogenous ABA treatments, but no obvious differences were observed between the transgenic lines and wild type (S4 Fig and S5 Fig).

The fourth sentence of the *AmDREB3* may play important regulatory roles in plant tolerance to drought, salt, and heat stressors subsection of the Discussion is incorrect. The correct sentence is: Furthermore, overexpressing *AmDREB3* in Arabidopsis conferred tolerance against drought, salt, and heat stressors (Figs 4, 5, 7 and 9), likely by activating or up-regulating the expression of some stress-inducible genes (Fig 10), but had no obvious effects on cold tolerance and ABA sensitivity (S4 Fig and S5 Fig).

The fourth sentence of the Constitutive expression of *AmDREB3* induced the transcription of stress-inducible genes in transgenic Arabidopsis subsection of the Results is incorrect. The correct sentence is: Here, we found that the transcript levels of all these genes were increased to between 1.7- and 9.0-fold in transgenic lines compared to those in wild type under normal conditions (Fig 10).
