# Supplementary figures and images for: Constitutive expression of an A-5 subgroup member in the DREB transcription factor subfamily from Ammopiptanthus mongolicus enhanced abiotic stress tolerance and anthocyanin accumulation in transgenic Arabidopsis
Source: PLoS One. 2019 Oct 23;14(10):e0224296. doi: 10.1371/journal.pone.0224296 (PMC6808444; doi:10.1371/journal.pone.0224296)

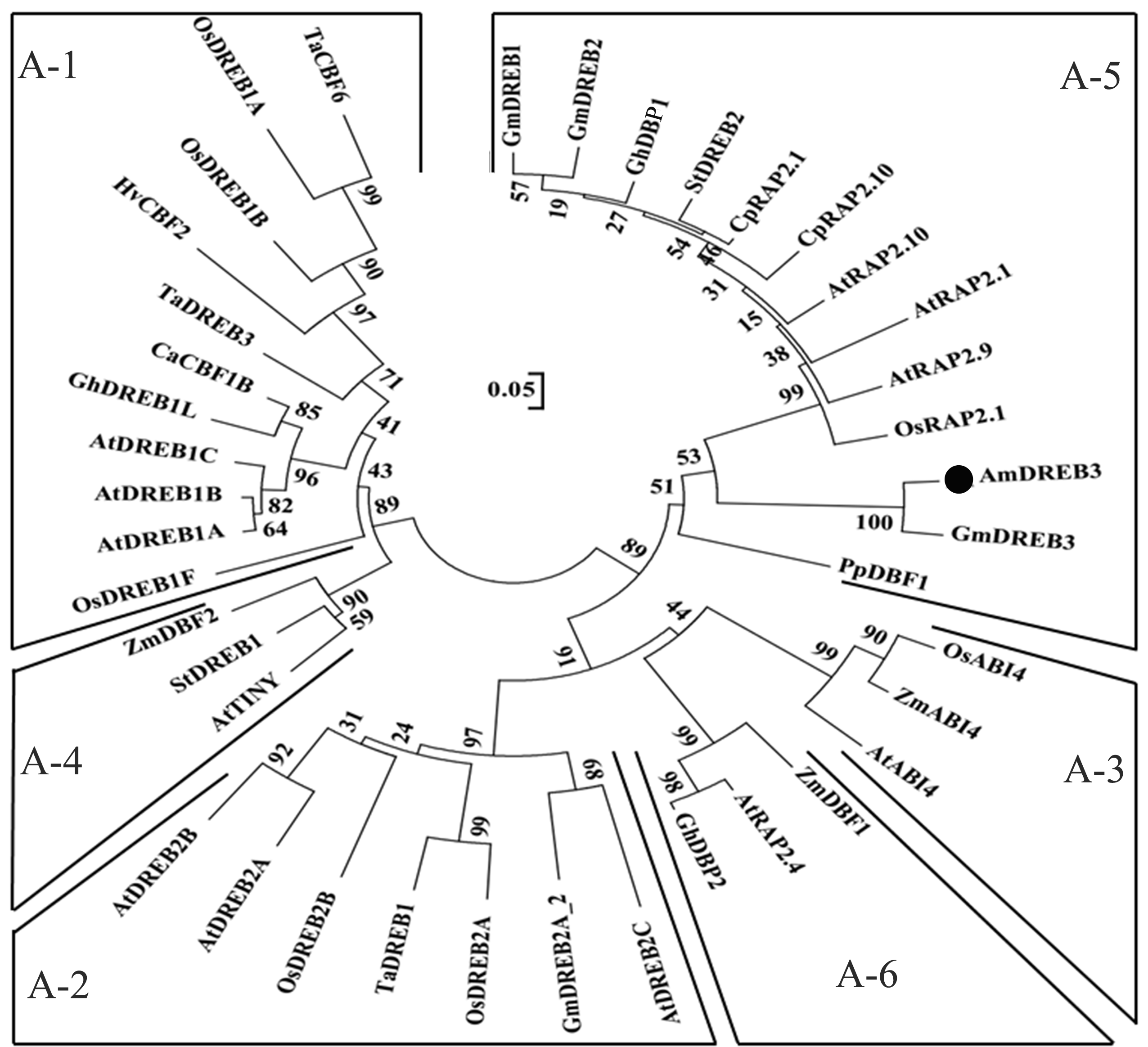

Supplement: S1 Fig — (TIF) [file pone.0224296.s001.tif]

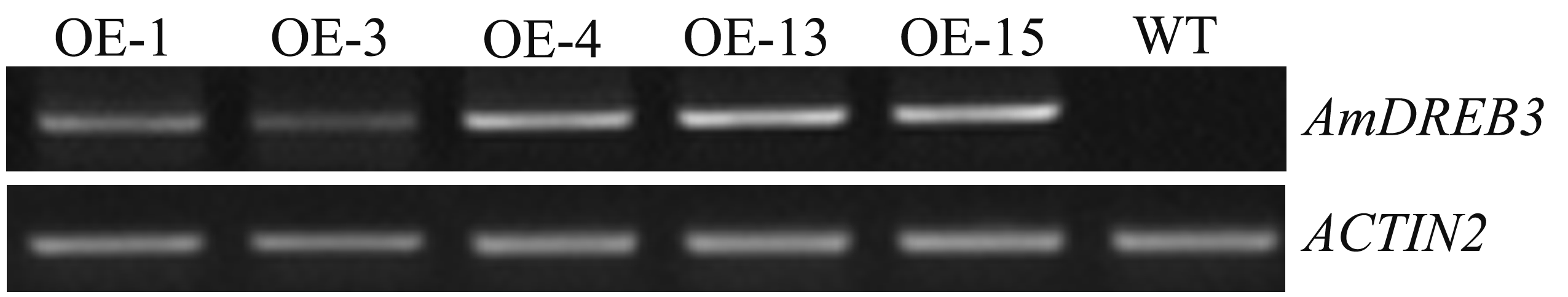

Supplement: S2 Fig — (TIF) [file pone.0224296.s002.tif]

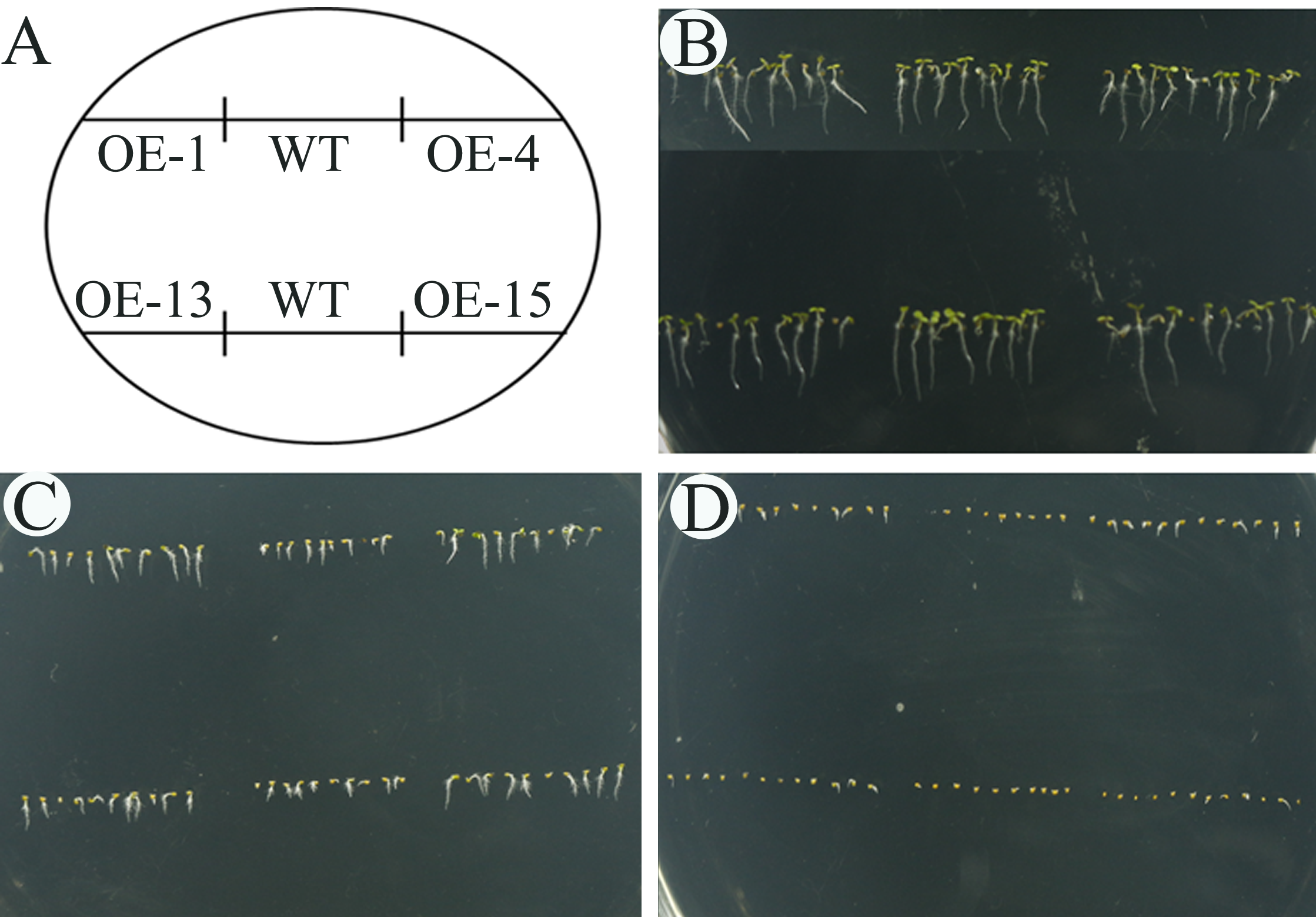

Supplement: S3 Fig — (TIF) [file pone.0224296.s003.tif]

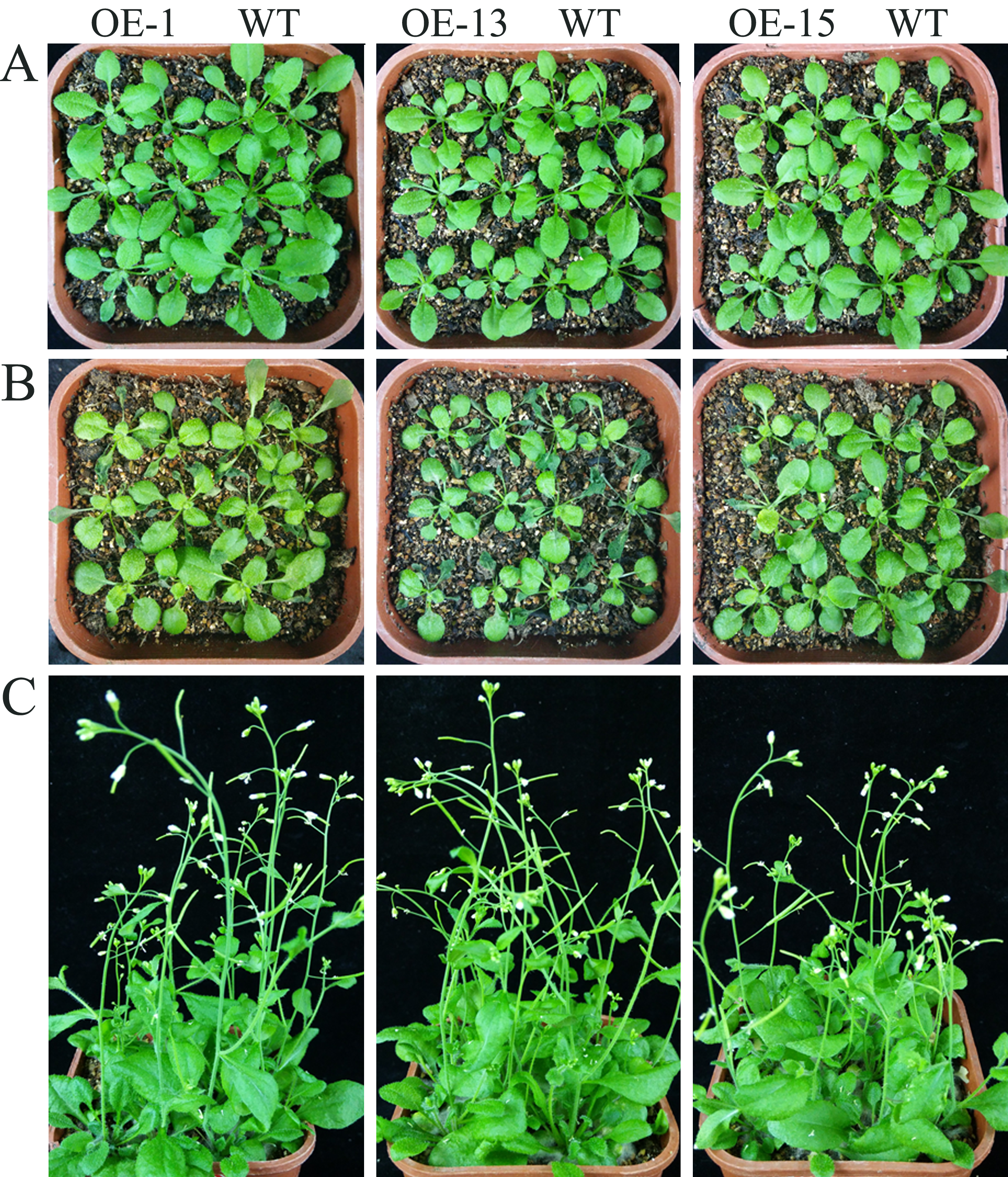

Supplement: S4 Fig — (TIF) [file pone.0224296.s004.tif]

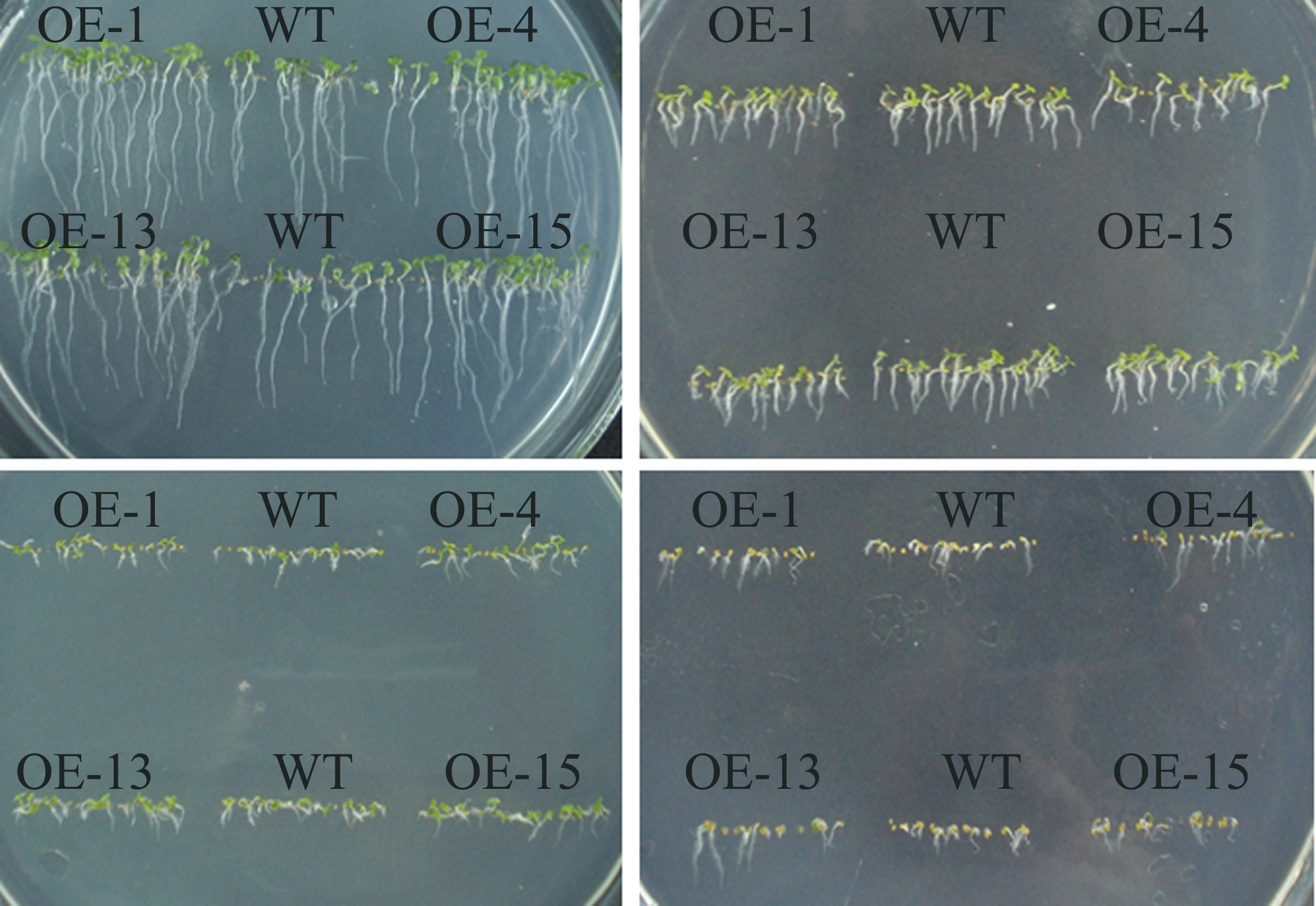

Supplement: S5 Fig — (TIF) [file pone.0224296.s005.tif]
