# Supplementary material for: Constitutive expression of an A-5 subgroup member in the DREB transcription factor subfamily from Ammopiptanthus mongolicus enhanced abiotic stress tolerance and anthocyanin accumulation in transgenic Arabidopsis
Source: PLoS One. 2019 Oct 23;14(10):e0224296. doi: 10.1371/journal.pone.0224296 (PMC6808444; doi:10.1371/journal.pone.0224296)
Supplement: S1 Table — (DOCX) [file pone.0224296.s006.docx]

S1 Table. List of primers used in this study

| Application | Reverse primer | Forward primer | Gene/primer name |
| --- | --- | --- | --- |
| Expression analysis | TTCCCCTTAGAAATTCCACAAG | GTCTCCGTCCCCTTCTCTATC | *AmDREB3-1* |
| Coding region amplification | TGGGTCACCCCTTAGAAATTCCAC | TAAGATCTATGGTGAAGCTCTCGAGCAT | *AmDREB3-2* |
| Subcellular localization | TTCCCGGGGAAATTCCACAAGAA | GTCTAGAAAATGGTGAAGCTCTCGA | *AmDREB3-3* |
| Transcription activity | CCCCGGGCCTTATCCTCTCCGTTAC | CGAATTCAAAAAAAACCTTACAACG | *AmDREB3-4* |
| Expression analysis | TAAACAAACCACAACGGCTC | TGGGTGAGGGTATGGAAGA | *AmTUB* |
| Expression analysis | CGATGCTGCCTTCTCGGTAGAG | GACGAGTCAGGAGCTGAGCTG | *AtRD29A* |
| Expression analysis | CACTTCCACCTCCTTTGTAGCCG | CCGACGGGAACTCATGATCAGTTC | *AtRD29B* |
| Expression analysis | CAGCGAATGTCCCACTCCCAC | GAAGCTCCCAGGACACCACGAC | *AtCOR47* |
| Expression analysis | CCGTAGCCACCAGCATCATA | CGTCTTACCAGAACCGTCCA | *AtRAB18* |
| Expression analysis | GGATGGGAATGTCCTGATGG | GTTCCACAACGCCAGCAC | *AtP5CS1* |
| Expression analysis | GAGCAGGCGCAGTAACGGTAAC | ACGGAATCAGAAAGACGGAGCAT | *AtHSFA4c* |
| Expression analysis | GTGGCGGTAGCTTCTTGATAACA | TCCGGCGAAAGTAATAACGAG | *AtHSP17.4-CIII* |
| Expression analysis | TCATACGGTCAGCGATACCTGAG | TGGTCGTACAACCGGTATTGTGC | *Actin2* |
|  | ATCCTCTGGCGACAGTTGGGTA |  | *AmDREB3-5-1* |
|  | TCACTCTGCTCCTCCTTGTCCTTT |  | *AmDREB3-5-2* |
|  | GATAGTCACGGTCAAAGGTGGAGATA |  | *AmDREB3-5-3* |

The primer pairs for 13 selected genes were shown.
