# Supplementary material for: Constitutive expression of an A-5 subgroup member in the DREB transcription factor subfamily from Ammopiptanthus mongolicus enhanced abiotic stress tolerance and anthocyanin accumulation in transgenic Arabidopsis
Source: PLoS One. 2019 Oct 23;14(10):e0224296. doi: 10.1371/journal.pone.0224296 (PMC6808444; doi:10.1371/journal.pone.0224296)
Supplement: S2 Table — (DOCX) [file pone.0224296.s007.docx]

S2 Table. DREB proteins used in the construction of the phylogenetic tree and their accession numbers

| Species | DREB protein | Accession Number |
| --- | --- | --- |
| *Arabidopsis thaliana* | AtDREB1C | BAA33436 |
| *Arabidopsis thaliana* | AtDREB1B | BAA33435 |
| *Arabidopsis thaliana* | AtDREB1A | BAA33434 |
| *Arabidopsis thaliana* | AtDREB2B | BAA36706 |
| *Arabidopsis thaliana* | AtDREB2A | BAA36705 |
| *Arabidopsis thaliana* | AtDREB2C | NP_565929 |
| *Arabidopsis thaliana* | AtABI4 | AF040959 |
| *Arabidopsis thaliana* | AtTINY | CAA64359 |
| *Arabidopsis thaliana* | AtRAP2.1 | NP_564496 |
| *Arabidopsis thaliana* | AtRAP2.9 | OAO89368.1 |
| *Arabidopsis thaliana* | AtRAP2.10 | NM_119854 |
| *Arabidopsis thaliana* | AtRAP2.4 | AEE36065.1 |
| *Oryza sativa* | OsDREB1F | AAX23723 |
| *Oryza sativa* | OsDREB1A | AF300970 |
| *Oryza sativa* | OsDREB1B | AF300972 |
| *Oryza sativa* | OsDREB2B | Q5W6R4.2 |
| *Oryza sativa* | OsDREB2A | AF300971 |
| *Oryza sativa* | OsABI4 | C7J2Z1.1 |
| *Oryza sativa* | OsRAP2.1 | XM_550356 |
| *Glycine max* | GmDREB2A-2 | AFU35563 |
| *Glycine max* | GmDREB1 | AF514908 |
| *Glycine max* | GmDREB2 | DQ208968 |
| *Glycine max* | GmDREB3 | DQ208969 |
| *Zea mays* | ZmABI4 | AY125490 |
| *Zea mays* | ZmDBF1 | AF493800 |
| *Zea mays* | ZmDBF2 | AF493799 |
| *Solanum tuberosum* | StDREB1 | JN125862 |
| *Solanum tuberosum* | StDREB2 | JN125858 |
| *Gossypium hirsutum* | GhDREB1L | ABD65473.1 |
| *Gossypium hirsutum* | GhDBP1 | AY174160 |
| *Gossypium hirsutum* | GhDBF2 | AY619718 |
| *Carica papaya* | CpRAP2.1 | KU065116 |
| *Carica papaya* | CpRAP2.10 | KU065117 |
| *Triticum aestivum* | TaDREB1 | AAL01124 |
| *Triticum aestivum* | TaCBF6 | AAX28964 |
| *Triticum aestivum* | TaDREB3 | ABC86564.1 |
| *Hordeum vulgaresubsp* | HvCBF2 | AAM13419 |
| *Capsicum annuum* | CaCBF1B | AAQ88400; |
| *Physcomitrella patens* | PpDBF1 | DQ202211 |
| *Populus euphratic* | PeDREB2a | ABU86872.1 |
| *Syntrichia caninervis* | ScDREB8 | AMT92116.1 |
| *Malus sieversii* Roem | MsDREBA5 | AFM84627.1 |
