# Supplementary material for: Constitutive expression of an A-5 subgroup member in the DREB transcription factor subfamily from Ammopiptanthus mongolicus enhanced abiotic stress tolerance and anthocyanin accumulation in transgenic Arabidopsis
Source: PLoS One. 2019 Oct 23;14(10):e0224296. doi: 10.1371/journal.pone.0224296 (PMC6808444; doi:10.1371/journal.pone.0224296)
Supplement: S3 Table — (DOCX) [file pone.0224296.s008.docx]

S3 Table. The sequence of *AmDREB3* promoter region.

TTGGGGACGACGGCAGTGATTCGAGCTCGGTACCCGGGGATCCTCTAGAGATTGATAGTCACGGTCAAAGGTGGAGATACAAAAACAAAAACAAAAAATAAATGTTTTATGATTTGTGAAATAATAATAGTAGTAGTATTTGTTCGGGTGGTGCTGACGGCTGAGTGCTGAGGTGAAGAATGTTTGTCGTTGGCTGATGATGTTGTTTGTGTTGGTGTTTGATGGGGTAGGGTTTGCGGCCCTATATATATATATATTCTCCCTCCCGATCTATATATAAGCAAAAATATATATAAATTTTTTGGACTCAAATATAAGCAAAAGTCAATCAAATCAATC.TTATTTAATGCTAGTGTTTCAGAAATACCATTTGCTTAATTGATTTTTTTTATTTTTTCAAGAGCATTATATACTCTAAAGATACAACTTTCAATACACTCCCAAGAAAGGGTAGTTTAGTAATCATAACTTATTTTTTTACATAAATTAAAACAATTAAACTAATATAACTAACTTTTTTAATATGTATAAAATTATTTTTTTTTTTATTTATATATGTATATACGGAGTAGTATATACTCCTGAGAATGTGGTGAGGTATGTGAGTATGAGTGTGGATTTGACTGCTAAAGCGGGTAAACAAGTTTGAAGGTCAACACTCTCGAGGCGCGTCCATTGAGGTTAGGAATCCATAATACTTGCTCTGCCTTCTACACGTGGTAGTGACGCGTCGCCGCTACGCGTTGTAACAGGTCACATTGTTGGACCACGTGCGTCTTTTAATCCACGTGAACTTACATTGCAAAACGGTTTCCACTTTGGAAAATGTTCTTCTTATCCAACTCCCGAATAACTACTGGATATTAGACATGTTCAATGTATATTTATAGATGCGTATAGATATAGAGTTTAATTGGTTTGCATTAAAAAGTGCATTCCCTCTTATGAATATTATAAACAAAAAAATTTTAAAATTTTTGATCGAATATATGAGTAAAAGATAATTACTTCCTCCTATTTAGTAACATTATTCATAAAATATCATTTTCATTTATTACCAATTTTATTTTTCAATAACTCATTTTATTTCAAGTCTCAAGTCCTTTTATAAATGATATTTTAAATAATAGATCTAATTTTTT**A**ACAATATTTAATTAGATTAATCAACTTTCTTAATATGTGTGTTCCATTAAATTTTTCTTTATAATACTGATCGGATGGAGTACTTAACTTCATATCCATAAATTTTAAGATAGTTAAGATAGGGATTAAACATTTTTAATCATTGATTGACAATGTAAAAAATCTTTATATTCATAGTGCATATTATTATTATTATTTCACAAATCATAAAACATTTGTTTTTTGTTTTTGTATCTCCACCTTTGACCGTGACTATCGGATACAAAAAAAACCTTACAACGTATGAAAAATGGTGAAGCTCTCGAGCATG
